# Preparation of modified Jiuzao glutelin isolate with carboxymethyl chitosan by ultrasound-stirring assisted Maillard reaction and its protective effect of loading resveratrol/quercetin in nano-emulsion

**DOI:** 10.1016/j.ultsonch.2022.106094

**Published:** 2022-07-12

**Authors:** Yunsong Jiang, Kai Zang, Jinyuan Sun, Xin-an Zeng, Hehe Li, Charles Brennan, Mingquan Huang, Ling Xu

**Affiliations:** a Key Laboratory of Brewing Molecular Engineering of China Light Industry, Beijing Technology and Business University, Beijing 100048, People’s Republic of China; b School of Food Science and Engineering, South China University of Technology, Guangzhou, People’s Republic of China; c School of Science, RMIT, Melbourne, VIC 3000, Australia; d Technology Center of Bandaojing Co. Ltd., Zibo, Shandong 256300, People’s Republic of China

**Keywords:** JGI, Jiuzao glutelin isolate, RES, resveratrol, QUE, quercetin, CTS, carboxymethyl chitosan, UTSA-MR, ultrasound-stirring assisted Maillard reaction, CTS-JGI-0.5, CTS-JGI-1, CTS-JGI-2, and CTS-JGI-4, CTS: JGI (0.5:1, 1:1, 2:1, and 4:1, w/w), CBC, cholesterol-binding capacity, MCI, micellar cholesterol inhibition, BAC, bile acid-binding capacity, AIA, α-amylase inhibitory activity, RES/QUE-CTS-JGI-2-O/W-NE, RES/QUE in CTS-JGI-2 stabilized oil-in-water nano-emulsion, RES/QUE-JGI-O/W-NE, native JGI formed oil-in-water nano-emulsion loaded with RES and QUE, EE, encapsulation efficiency, LC, loading capacity, RR, release rate, BAY, bioavailability, CTSU, CTS structure unit, CTS-JGI conjugates, Ultrasound-stirring, Emulsifying and function properties, Nano-emulsion, Resveratrol/quercetin, Bioavailability

## Abstract

Jiuzao glutelin isolate (JGI) was reported to possess interface and functional properties. To enhance the stability and properties of JGI, conjugation between JGI and carboxymethyl chitosan (CTS) through ultrasound-stirring assisted Maillard reaction (UTSA-MR) was investigated and optimized. The changes of molecular distribution, secondary structure, morphology, and amino acid composition of JGI were detected after conjugation with CTS. The solubility, foaming property and stability, viscosity, and thermal stability of four conjugates (CTS-JGI, with weight ratios of 0.5:1, 1:1, 2:1, and 4:1) were significantly increased compared to native JGI. Under the optimal glycation, the conjugate (CTS/JGI, 2:1, w/w; CTS-JGI-2) exhibited the best emulsifying ability and stability against NaCl solution, *in vitro* antioxidant activity, and cholesterol-lowering ability. CTS-JGI-2 stabilized oil-in-water nano-emulsion improved resveratrol (RES) and quercetin (QUE) encapsulation efficiency (80.96% for RES and 93.13% for QUE) and stability during the simulated digestion process (73.23% for RES and 77.94% for QUE) due to the connection through hydrogen bonds, pi-anion, pi-sigma, and donors between CTS-JGI and RES/QUE. Taken together, the modification of JGI by conjugating with CTS through UTSA-MR could be an excellent method to improve the functional properties of JGI. CTS-JGI-2 is a potential conjugate with functions that can be used to encapsulate functional substances in the stabilized nano-emulsion.

## Highlights

• Jiuzao glutelin isolate (JGI) was glycated with carboxymethyl chitosan (CTS) through ultrasound-stirring assistant Maillard reaction.
• JGI’s stability and emulsifying properties against electrolyte in the nano-emulsion system were improved after conjugation.
• CTS-JGI-2 exhibited better *in vitro* activities than JGI.
• CTS-JGI-2 stabilized nano-emulsion improved the bioaccessibilty of resveratrol and quercetin.

## Graphical abstract

**Figure:**
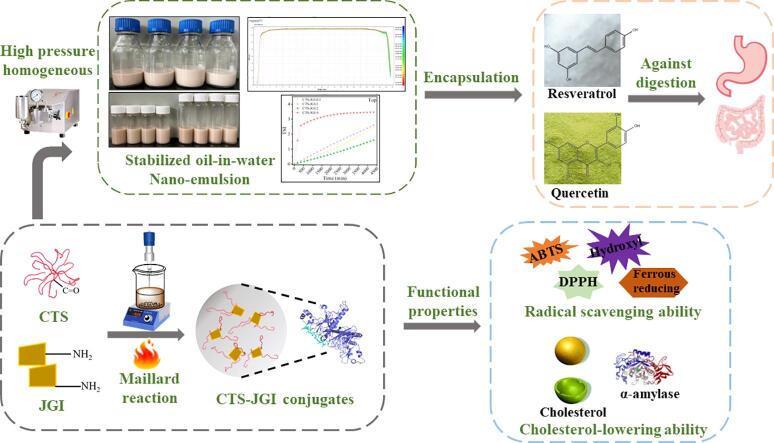

## 1 Introduction

Baijiu is the Chinese national liquor and is popular with people of different ages. Jiuzao is the solid by-product after baijiu distillation [16]. In recent years, baijiu production has shown an increasing trend, and more content of Jiuzao has been produced. The raw materials of baijiu distillation are mainly sorghum, accompanied by rice, wheat, corn, barley, and glutinous rice [41]. These raw materials have a high protein content. Some proteins in the raw materials are the primary nutrients for microorganisms in the fermentation process [18]. However, the proteins not utilized by microorganisms remain in Jiuzao after the distillation process due to their high boiling points. Currently, the kafirin and glutelin in Jiuzao have been studied in the literatures [18], [55]. Although the content of kafirin is higher than glutelin in sorghum, glutelin possesses higher essential amino acids that the body needs [6]. In addition, our previous study has extracted and purified Jiuzao glutelin isolate (JGI) and identified its structure by nano ultra-performance liquid chromatography-mass spectrum/mass spectrum [18]. High interfacial properties (such as foamability, foam stability, and water and oil retention properties) and functional properties (such as free radical scavenging ability and ACE inhibitory activity) of JGI were found. Therefore, JGI has higher research and utilization values.

Proteins are a class of substances that has a variety of functions. Animal and plant proteins are the two most common protein sources. [11]. Compared with animal protein, plant protein is mainly obtained from crops and is derived from by-products of food processing [48]. Compared with the acquisition of animal protein, there is a wider variety of plant protein. Using by-products can also reduce the pollution they cause to the environment. Plant protein with sensory quality similar to specific animal protein is mainly processed from raw plant materials, which not only meets the people's pursuit of taste but also provides the nutritional requirement of reducing saturated fatty acid and cholesterol intake. Plant protein is an active substance that plays a vital role in sustaining normal metabolism and is essential in the body [2]. Plant protein can be utilized as a carrier for active ingredients [40]. However, when proteins are influenced by external conditions (such as pH changes, temperature, acidity, alkalinity, ion concentration, and others.), their original functional properties are also altered [1]. Therefore, it is imperative to improve the stability of proteins.

Maillard reaction occurs between amino groups of proteins and peptides with carbonyl groups of polysaccharides [24]. Although the degree of Maillard reaction is difficult to control, relevant studies have demonstrated that protein stability and functional properties can be significantly improved by combining with polysaccharides at the optimal Maillard reaction degree. Klinchongkon conjugated whey protein with subcritical-water hydrolyzed pectin and found that the conjugates exhibited better emulsifying properties than whey protein [20]. Wen prepared soy protein isolate-lentinan conjugates through Maillard reaction by slit divergent ultrasonic-assisted wet heating method and found better functional properties of conjugates [45]. As a new environmental protection technology, ultrasound has been widely used in the food industry. The high temperature and pressure caused by the ultrasonic process provide the conditions for the Maillard reaction. Ultrasound has been applied to accelerate the Maillard reaction, resulting in less hazardous ingredient formation and increasing the conjugate functions. Zhang utilized the ultrasound-assisted wet-heating method to produce whey protein-flaxseed gum product and found better physicochemical properties and encapsulation efficiency of astaxanthin [53]. Jiang also prepared pea protein-inulin conjugates with higher surface load and thicker interfacial layers via ultrasound Maillard reaction to improve the sensory characteristics and emulsifying ability [15]. Yang used energy-divergent type ultrasound to promote the glycosylation of protein-hydrolysate from grass carp to improve the flavor of the products [52].

Chitosan is the product of chitin deacetylation. Chitosan has a variety of physiological functions such as biodegradability, biocompatibility, non-toxicity, antibacterial, anti-cancer, lipid-lowering, and immune enhancement. Chitosan is used in various industries, including the food industry, drug sustained-release materials, gene transduction carriers, biological medical, and daily chemical industries [27], [43]. Chitosan has many unique properties such as biodegradability, cell affinity, and biological effects [30]. In Xu’s and Du’s studies, chitosan was conjugated with soybean protein isolate (SPI) to improve the SPI’s emulsifying activity and stability [9], [50].

Resveratrol (RES, a non-flavonoid polyphenol compound) and quercetin (QUE, a flavonol compound) are proved to possess functional properties, such as antioxidant, anti-inflammatory, anti-cancer, and cardiovascular protection. However, their applications in the food and pharmaceutical industries are limited due to their instability, low water solubility, and poor oral bioavailability. Therefore, many delivery strategies, for instance, liposomes [5], liquid self-micro emulsifying drugs [13], and polymeric micelles [10] have been studied to overcome these limitations. Emulsions have a wide range of applications in the food field, including homogeneous milk, cream, and seasonings [4]. Nano-emulsion is a new type of emulsion with the advantages of using less emulsifier, having no surface-active additives, and being stable [18]. Nano-emulsion can be used to encapsulate and protect the release of hydrophobic natural products in the delivery system, considering the enhancement of water solubility, thermal stability, gastrointestinal stability, and bioavailability because of its versatile technical functions and biological properties [7].

Carboxymethyl chitosan (CTS) has a better water solubility than chitosan. Currently, no study attempts to prepare Maillard products using JGI and CTS as raw materials to explore the improvement of their physicochemical properties and utilization in the delivery systems for RES and QUE. The purpose of this study was to conjugate JGI with CTS via the ultrasound-stirring assisted Maillard reaction (UTSA-MR) to improve the physicochemical, interfacial, and functional properties as well as to explore its encapsulation effect on RES and QUE in oil-in-water nano-emulsions. The main contents include 1) optimization of UTSA-MR conditions; 2) determination of interfacial properties and JGI structure changes of Maillard products; 3) measurement of Maillard products stabilization on oil-in-water nano-emulsions; 4) investigation of functional properties of Maillard products (*in vitro* antioxidant and cholesterol-lowering effects); 5) exploration of RES and QUE encapsulation ability of conjugate stabilized nano-emulsion. The above research aims to broaden the application of JGI in food emulsions and functional foods industry, improve the utilization of JGI, and reduce the environmental pollution caused by Jiuzao’s untimely treatment.

## 2 Materials and methods

### 2.1 Materials and reagents

Jiuzao of strong-flavor type (after three times of distillation; main material composition, sorghum, wheat, corn, rice, sticky rice, and millet; auxiliary material composition, rice husk) was provided by Bandaojing Liquor Co., ltd. (Zibo, China). The identified JGI (Table S1) was obtained according to our previous method [18]. CTS, RES, and QUE (purity > 98 %) were purchased from Macklin Biochemical Co., ltd (Shanghai, China). Other materials and reagents were exhibited in the Supplementary Material.

### 2.2 Preparation of carboxymethyl chitosan-Jiuzao glutelin isolate (CTS-JGI) conjugates by ultrasound-stirring assisted Maillard reaction (UTSA-MR)

CTS-JGI conjugates were prepared via the UTSA-MR in a wet-heating system following a method [26] with some modifications. First, CTS and JGI were dissolved in ultrapure water to obtain a concentration of 10 mg/mL solutions. The solutions were stirred for 3 h at 25 ℃. The dissolved CTS and JGI solutions were mixed to obtain a series ratio of 0.5:1, 1:1, 2:1, and 4:1 (CTS/JGI, w/w), which were expressed as CTS-JGI-0.5, CTS-JGI-1, CTS-JGI-2, and CTS-JGI-4. The mixture solutions were stored at 4 ℃ overnight to obtain complete hydration. Next, the mixture was transferred to glass bottles and capped to prevent water evaporation during the reaction. The pH of the CTS-JGI solutions was adjusted to 7.0 and 11.0. The sealed samples were incubated and stirred in a magnetic stirrer (IKA, RCT basic, Baden-Wuerttemberg, German) at 90 ℃ along with the treatment of ultrasound (400 W, 10 s on and 10 s off at a frequency of 25 kHz) using an ultrasound cell disruptor (Shanghai Huxi Industrial Co., ltd) with a Φ6 horn. The samples were collected at different times (0, 15, 30, 60, 90, 120, and 180 min). The collected samples were immediately cooled in an ice-water bath to stop reaction and stored at 4 ℃ for further experiments. JGI treated without UTS was used as a control.

### 2.3 Determination of the pH changes, browning index (BI), A_294_, grafting degree (GD), and surface disulfide bond content

The methods of the pH changes, BI, A_294_, GD, and surface disulfide bond content was exhibited in the Supplementary material.

### 2.4 Structure properties

#### 2.4.1 Molecular distribution analysis by high-performance size-exclusion chromatography (HPSEC)

CTS-JGIs with the best Maillard reaction degree obtained in each CTS/JGI ratio were used for the subsequent investigation.

HPSEC was performed to investigate the molecular size distributions of CTS-JGIs. The analysis was conducted using an Agilent HPLC instrument (model 1260, Santa Clara, CA, USA) equipped with a TSKgel SW2000 column (separation range 15–150 kDa, TOSOH, Tokyo, Japan). 50 μL of CTS-JGI (0.67 mg/mL) solution was injected by an autosampler. The same mobile phase and elution procedure in a previous study [18] was used to analyze the molecular distribution. The chromatograms were recorded at 214 and 280 nm.

#### 2.4.2 Ultraviolet (UV)-visible spectroscopy

The sample solutions (0.1 mg/mL) were prepared with ultrapure water. The UV–visible spectra of CTS-JGIs were recorded from 240 to 320 nm at room temperature (25 ℃) using a UV–vis spectrophotometer UV-2700 (Shimadzu, Kyoto, Japan) to measure the structure changes of CTS-JGIs.

#### 2.4.3 Fourier transform infrared (FT-IR) analysis

The samples were obtained after being freeze-dried using a lyophilizer (Christ, Alpha 1–4 LSC basic, Osterode, German). The FT-IR spectrum was recorded using a vector 33 IR spectrophotometer (Bruck, Ettingen, Germany) at a wavenumber of 4000–800 cm^−1^.

#### 2.4.4 Circular dichroism (CD) analysis

2.5 mg/mL of the sample solutions were prepared and filled in a precision quartz cell of 2.0-mm. The CD spectrum of CTS-JGI was recorded using a J-815CD Spectrometer (JASCO, Tokyo, Japan) at a wavelength of 195 to 300 nm. The secondary structure of CTS-JGI was analyzed by the Dicroprot software.

#### 2.4.5 Determination of surface hydrophobicity (H_0_)

The samples (0.01, 0.05, 0.1, 0.15, and 0.2 mg/mL) were prepared with 10 mM pH 7.0 PBS solution. The method of H_0_ measurement was shown in the Supplementary Material.

#### 2.4.6 Scanning electron microscopy

The samples adhered to a conductive paste. After gold spraying, the apparent morphology of the conjugates was recorded at 3.0 kV using a scanning electron microscope (SEM) (FEI Quanta 250 FEG, FEI Inc., QR, USA).

#### 2.4.7 Amino acid composition analysis

The amino acid compositions of the samples were analyzed by an automatic amino-acid analyzer (HITACHI, L-8900, Tokyo, Japan). First, the samples were hydrolyzed by 5.7 M HCl at 110 ℃ for 12 h. Then, 1 mL of hydrolysate was evaporated in rotation. Finally, the hydrolysate was redissolved in 0.02 M HCl and filtered through a 0.22 μm membrane.

### 2.5 Physicochemical properties

The solubility, foaming property and stability, emulsification activity index (EAI), emulsification stability index (ESI), viscosity, and thermal stability methods were exhibited in the Supplementary Material.

### 2.6 Physical stability of oil-in-water nano-emulsions during 28 days of storage

#### 2.6.1 Preparation of oil-in-water nano-emulsions

The soybean oil and CTS-JGI conjugates (5 mg/mL) were mixed equably at a ratio of 1:100 (v/v). The mixed solution was homogenized for 3 min at 15,000 rpm in an ice bath to obtain the coarse emulsion. The oil-in-water nano-emulsion was obtained by homogenizing the coarse emulsion circularly using a high-pressure homogenizer (APV 2000, Beijing, China) at 750 bars for 6 min. The prepared oil-in-water nano-emulsions were used for subsequent analysis.

#### 2.6.2 Particle size, polydispersity index (PDI), and zeta-potential measurement

The particle size, PDI, and zeta-potential of the emulsions were directly measured using a Malvern Mastersizer 3000 (Malvern Panalytical Inc., Malvern City, UK) at 1, 7, 14, 21, and 28 days.

#### 2.6.3 Backscattering intensity (BSI) and Turbiscan scan index (TSI) measurement

The BSI and TSI of conjugates were evaluated via acceleration testing at 40 ℃ using a multiple light scattering spectroscopy (Turbiscan Tower, Formulaction, Toulouse, France). The prepared emulsions were transferred to 20 mL glass tubes. The glass tubes were scanned from the bottom to the top for 3 d, measuring BSI and TSI to inspect the destabilization mechanism.

#### 2.6.4 Stability of emulsions against NaCl

NaCl solution (5 M) was added to the emulsions to achieve the final concentrations of 50 and 150 mM in the emulsions. The particle size and PDI of the emulsions were directly measured at 1, 7, 14, 21, and 28 days.

### 2.7 Functional properties

#### 2.7.1 *In vitro* antioxidant activity

The CTS-JGI solutions were prepared with 0.125, 0.25, 0.625, 0.9375, and 1.25 mg/mL concentrations. ABTS, DPPH, hydroxyl radical scavenging, and ferrous reducing abilities were evaluated according to the instruction manufacturers.

#### 2.7.2 Cholesterol-lowering activities

The cholesterol-binding capacity (CBC) of JGI and CTS-JGI-2 were measured according to Zhu’s method [56] with some modifications. The specific method was shown in the Supplementary Material.

The micellar cholesterol inhibition (MCI) of the samples was measured according to the method described by [46] with minor modifications. The specific method was stated in the Supplementary Material.

The specific method of bile acid-binding capacity (BAC) of JGI and CTS-JGI measurement was shown in the Supplementary Material.

α-amylase inhibitory activity (AIA) of the samples was determined according to a DNS assay kit instruction [23]. The specific method was exhibited in the Supplementary Material.

### 2.8 Protection effect of RES/QUE in CTS-JGI-2 stabilized oil-in-water nano-emulsion (RES/QUE-CTS-JGI-2-O/W-NE)

#### 2.8.1 Construction of RES/QUE-CTS-JGI-2-O/W-NE

Weighted RES and QUE were dissolved in dimethyl sulfoxide (DMSO) to achieve a concentration of 100 μM. Then, the prepared solution was mixed with CTS-JGI-2 solution (10 mg/mL, pH 8.5) at room temperature for 4 h to integrate fully. Afterward, the O/W-NE were prepared with the same method in Section 2.6.1. Native JGI formed oil-in-water nano-emulsion loaded with RES and QUE (RES/QUE-JGI-O/W-NE) was used as the control.

#### 2.8.2 Encapsulation efficiency (EE) and loading capacity (LC)

The EE and LC of RES and QUE in the emulsions were measured by the method of [7] with slight modification. The RES/QUE-CTS-JGI-2-O/W-PE was diluted 50-fold with DMSO and centrifuged at 4,000 *g* for 15 min. Finally, the absorbance was measured at 320 nm and 360 nm, respectively. The EE and LC were calculated according to the following equations and compared to RES/QUE-JGI-O/W-NE:

(1) $$\mathrm{EE}\;\left(\%\right)=\left(\frac{A-B}{A}\right)\times 100\%$$

where A is the initial content (mg/mL) of RES and QUE, and B is the content of free RES and QUE content in the supernatant.

(2) $$\mathrm{LC}\;\left(\%\right)=\left(\frac{A-B}{W}\right)\times 100\%$$

where W is the weight (mg) of RES/QUE-CTS-JGI-2-O/W-NE or RES/QUE-JGI-O/W-NE.

#### 2.8.3 *In vitro* simulated digestion

20 mL of RES/QUE-CTS-JGI-2-O/W-NE was prepared for the *in vitro* simulated digestion assay. The method was determined according to our previous study [16] with slight modification; that is, the digestion of gastric and intestinal stages were 2 h. After digestion, 1 mL of the digesta was centrifuged for 20 min at 4 ℃ and 5,000 *g*. The supernatant was collected and diluted by DMSO (20-fold). The content of RES and QUE was measured by the method in Section 2.9.2. The release rate (RR) and bioavailability (BAY) were measured by the following equations and compared with RES/QUE-JGI-O/W-NE:

(3) $$\mathrm{RR}\;\left(\%\right)=\left(\frac{\mathrm{Content}\;of\;RES\;and\;QUE\;in\;the\;\sup erna\tan t}{\mathrm{Total}\;RES\;and\;QUE\;content\;in\;the\;digesta}\right)\times 100\%$$

(4) $$\mathrm{BAY}\;\left(\%\right)=\left(\frac{\mathrm{Content}\;of\;RES\;and\;QUE\;in\;the\;emulsion}{\mathrm{Total}\;RES\;and\;QUE\;content\;before\;digestion}\right)\times 100\%$$

#### 2.8.4 SEM observation of RES- and QUE-loaded CTS-JGI-2 powder

To investigate the connection of RES and QUE with CTS-JGI-2, SEM was used to observe the microstructure with the same protocol in Section 2.4.6.

#### 2.8.5 Prediction of the potential binding mechanism of RES and QUE with CTS-JGI-2

Molecular docking software AutoDock 4.2 (Scripps, CA, USA) was used to predict the docking sites of RES and QUE with CTS-JGI. Discovery Studio 2019 (BIOVIA, CA, USA) and PyMOL 2.5 (Schrodinger, DE, USA) were used to produce the docking graphs. The detail method and JGI macromolecule were the same as in our previous study [17], [18]. The CTS structure unit (CTSU) was obtained from http://www.chemspider.com/Default.aspx with a ChemSpider ID of 64780. RES and QUE were prepared using Chem3D Professional 16.0 (CambridgeSoft, MA, USA).

### 2.9 Statistical analysis

All measurements were performed on at least four freshly prepared samples and reported as mean and standard deviation. The results were analyzed using SPSS software (SPSS Inc., Chicago, II. USA) for one-way ANOVA analysis. The level of significance was determined at *p* < 0.05.

## 3 Results and discussion

### 3.1 Degree of Maillard reaction

Maillard reaction can improve the properties of proteins. However, overreaction also produces harmful substances, such as carcinogens [39]. Therefore, to control the degree of reaction, the overall reaction time was controlled at 180 min to optimize the experiment to avoid excessive reaction producing harmful substances in this study.

#### 3.1.1 pH changes

**Fig. 1:**
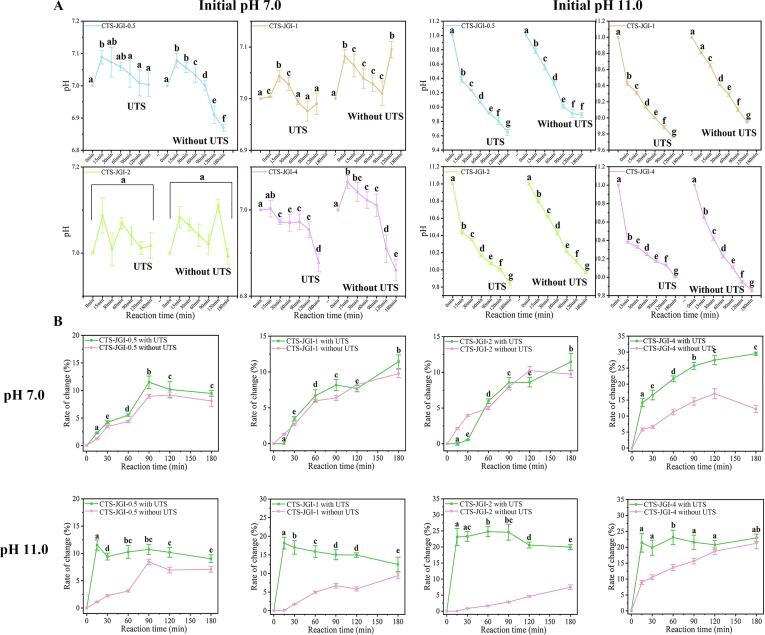
(A) pH changes during the reaction. (B) BI rate changes during the reaction. Different letters showed a significant difference at *p* < 0.05 in the UTS treated groups.

pH changes are shown in Fig. 1A. There was no noticeable change in pH when reacted in the neutral condition. pH showed an upward trend in CTS-JGI-0.5 and CTS-JGI-1conjuates during the first 1 h. This result is due to the opening of the JGI spatial structure after the reaction started, and the exposed amount of alkaline amino acids was more than the amount involved in the reaction ascribed to the low ratio of CTS. This increase did not occur in CTS-JGI-2 and CTS-JGI-4 conjugates, which proved that a high proportion of CTS could react rapidly with these alkaline amino acids. However, in the pH 11.0 condition, the pH value exhibited a significant (*p* < 0.05) decrease in the four conjugates. The pH changes in UTSA-MR are lower than untreated ones, indicating the reaction degree treated by UTS is higher than the traditional method. During the reaction, many alkaline amino acids were consumed when reacted with the carbonyl of CTS to produce hydroxymethyl furfural or furfural [19]. The pH of the solution cannot maintain in a high alkaline condition, so it dropped rapidly after the reaction started. As the reaction progressed and approached completion, the trend of pH decline slowed down. The above results proved that the Maillard reaction occurred between CTS and JGI.

#### 3.1.2 BI and A_294_

**Fig. 2:**
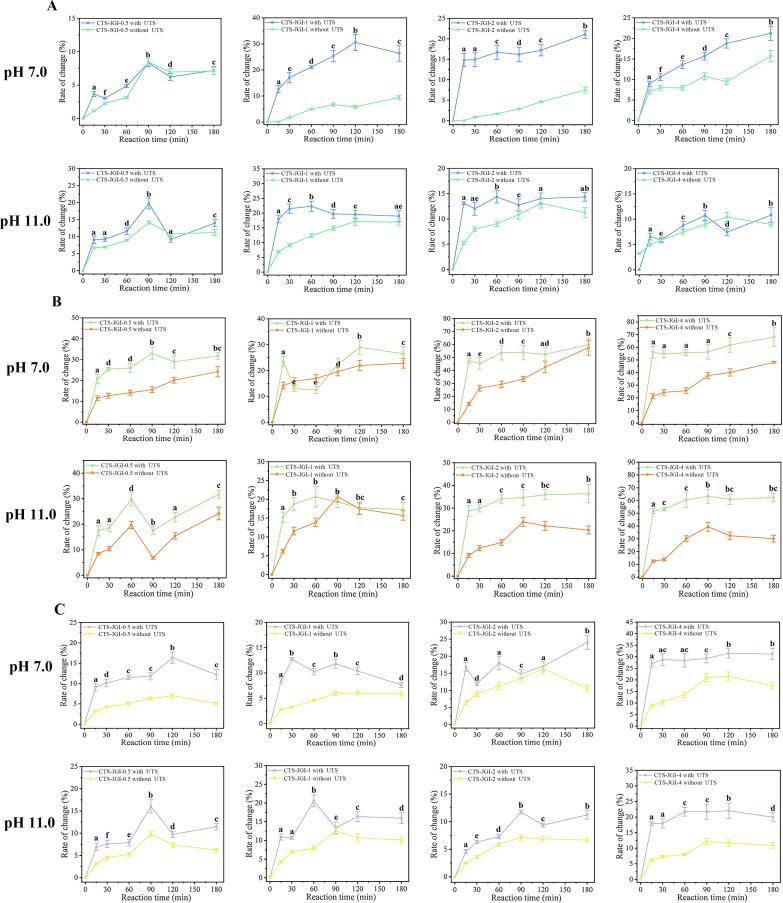
(A) Intermediate product changes during the reaction. (B) GD during the reaction. (C) Surface disulfide bond changes during the reaction. Different letters showed a significant difference at *p* < 0.05 in the UTS treated groups.

A color change accompanies with the Maillard reaction. Yellow and yellow/brown products are formed in the middle and final stages of the Maillard reaction, respectively [36]. Therefore, measuring the color change can reflect the progress of the reaction. The characteristic absorbance at 420 nm and 294 nm was measured (Fig. 1B and Fig. 2A). It could be seen that the absorbance at 420 nm and 294 nm in the pH 7.0 condition along with 294 nm in the pH 11.0 condition presented an upward trend when reacting. This result indicates that as the reaction proceeds, the content of colored substances gradually increases. However, a different trend appeared in the absorbance at 420 nm in alkaline conditions. The absorbance increased rapidly in the first 15 min and decreased subsequently. This phenomenon is caused by the decreased pH during the reaction process, which reduces the solubility of trace tannins and anthocyanins in JGI [18], causing different colors of tannins and anthocyanins to exhibit under different acid-base conditions [29]. The degree of color lightening caused by the decrease in solubility of tannins and anthocyanins is higher than the content of brown substances produced in the reaction. Thus, the absorbance value at 420 nm in the subsequent reaction decreases. The absorbance value under alkaline conditions is significantly higher than that under neutral conditions. One reason is that JGI has a high solubility under alkaline condition, which contributes to complete reaction. Thus, higher content of brown substances is formed. The other reason is that tannins and anthocyanins interfere with color.

#### 3.1.3 GD

The GD during the reaction was measured to investigate the degree of the Maillard reaction. The result is shown in Fig. 2B. Under neutral and alkaline conditions, GD shows an upward trend. With the increase of the CTS proportion, the GD value increased, indicating that the GD value was positively correlated with the amount of CTS added. Moreover, the GD between CTS and JGI is higher at pH 7.0 than at pH 11.0.

#### 3.1.4 Surface disulfide bond content

In addition, the content of surface disulfide bonds can reflect the changes in protein structure during the reaction process, which can indirectly reflect the degree of Maillard reaction [52]. The result is presented in Fig. 2C. As the reaction progressed, the surface disulfide bond content increased at both pH values. However, the reaction times to reach the maximum value were different among the four ratio reactions. Besides, comparing the reactions at pH 7.0 and pH 11.0, the surface disulfide bond content is significantly higher in the neutral environment than in the alkaline environment. This phenomenon indicates that the Maillard reaction proceeds to a higher degree in a neutral environment.

Based on the above four indicators, the BI, intermediate products, GD, and surface disulfide bond changes in UTSA-treated groups are higher than in untreated traditional reaction groups. This result proves that UTSA could accelerate the reaction rate by supporting more free amino groups of JGI and accelerating their contact with carbonyl radicals of CTS. Same results were also reported in Zhang and Jiang’s studies [18], [53]. In addition, four proportions of CTS-JGI conjugates under the condition of the highest degree of UTSA-MR were selected for subsequent experiments. The optimal conditions are CTS-JGI-0.5 reacted for 120 min at pH 7.0 (with a total change percent of 69.27 % in four indexes), CTS-JGI-1, CTS-JGI-2, and CTS-JGI-4 reacted for 180 min at pH 7.0 (with total change percent of 81.38 %, 116.48 %, and 144.54 %, respectively, in four indexes). This result proves that as the proportion of CTS elevates, the degree of reaction increases significantly.

### 3.2 CTS-JGI structure properties

#### 3.2.1 HPSEC analysis

**Fig. 3:**
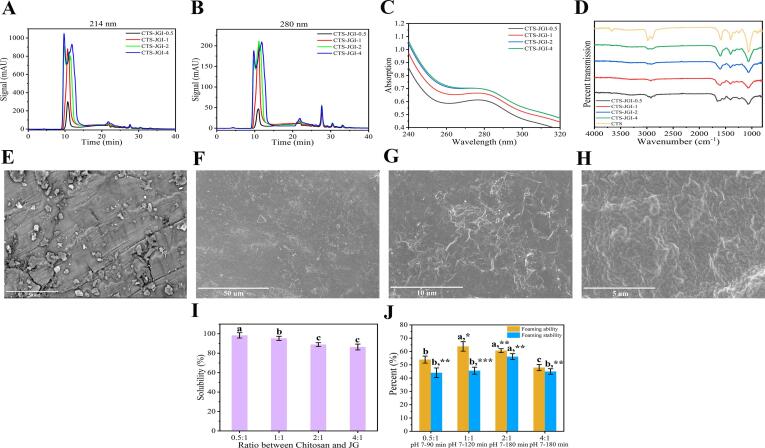
HPSEC profiles of the CTS-JGI conjugates at (A) 214 nm and (B) 280 nm. (C) UV spectrogram of the CTS-JGI conjugates. (D) FT-IR spectrogram of the CTS-JGI conjugates. (E) Morphology observation graphs of the heated JGI and (F-H) CTS-JGI conjugates at different resolutions. (I) The solubility of the CTS-JGI conjugates. (J) Foaming ability and stability of the CTS-JGI conjugates. Different letters showed a significant difference at *p* < 0.05. *, *p* < 0.05, **, *p* < 0.01, and ***, *p* < 0.001 means significant difference between JGI in the previous study and CTS-JGI conjugates.

To accurately analyze the changes in the molecular weight distribution of JGI after the reaction, HPSEC is used instead of SDS-PAGE to analyze whether new molecular weight peptides are generated during the reaction. The elution profiles of the CTS-JGI conjugates at 214 nm and 280 nm are shown in Fig. 3A and B. Large molecules are usually eluted before small molecules in HPSEC. Compared with the native JGI molecular distribution stated in the previous study [18]. The prominent peak of JGI at 10 min resolved into two peaks when conjugated with high CTS ratios (CTS-JGI-2 and CTS-JGI-4). This result was due to sufficient CTS-JGI reaction in CTS-JGI-2 and CTS-JGI-4 to unwind the spatial structure of JGI in the high molecular weight region, resulting in the two peaks at around 10 min. At the low molecular weight region (20–40 min), the peaks at 25 min and 28 min disappeared, while new peaks appeared at 27 min, 31 min, and 33 min. This result indicates a disappearance of peptides and the generation of peptides with new molecular weight during the Maillard reaction between JGI and CTS. The same phenomenon also appeared in Chen’s study [7].

#### 3.2.2 UV analysis

Proteins have an apparent UV absorption at 280 nm (on account of the presence of conjugated double bonds in tryptophan and tyrosine residues), so the UV absorption of CTS-JGI at 280 nm was determined. The result is shown in Fig. 3C. Blue shifts of the absorbance in CTS-JGI appeared compared to native JGI studied in the previous study [18], which may be ascribed to the change in solution polarity, pH, and the decrease in the degree of JGI conjugation as the reaction proceeds. A slight red shift appears in the UV absorption as the proportion of CTS increases. This red shift is caused by the increase in the proportion of CTS, which makes it react fully with JGI and significantly changes the spatial structure of JGI. In addition, the formation of organic compounds such as ketones and aldehydes during the reaction increases the polarity of the solution, which also contributes to the red shift phenomenon. The same phenomenon was also reported in Chen’s study that red shift appeared, indicating the fish scales peptides and xylose were cross-linked [8].

#### 3.2.3 FT-IR analysis

FT-IR is an effective method to explore the structures of proteins and polysaccharides. As shown in Fig. 3D, CTS possesses noticeable peaks at 2989.123 cm^−1^ and 2901.378 cm^−1^, an –NH angular deformation peak at 1592.913 cm^−1^, and a -C—O—C- peak at 1066.442 cm^−1^ [9]. JGI exhibits amino acid characteristic bands at 2924.87 cm^−1^ (C—H stretching), 1651.50 cm^−1^ (α-helix in amide I), 1532.52 cm^−1^, 1517.70 cm^−1^, 1453.07 cm^−1^ (N—H bond vibrations), and 1233.26 cm^−1^ (β-strand) [18]. After conjugation, the characteristic bands of CTS at 2989.123 cm^−1^, 2901.378 cm^−1^, 1592.913 cm^−1^ and JGI at 1532.52 cm^−1^, 1517.70 cm^−1^, and 1453.07 cm^−1^ disappeared. This result indicates that CTS-JGI electrostatic interaction occurred in four combinations of conjugates. Red shifts appeared at 2925.996 cm^−1^, 1655.569 cm^−1^, 1484.437 cm^−1^, 1405.370 cm^−1^, 1247.718 cm^−1^, 1194.685 cm^−1^, and 1064.996 cm^−1^. These shifts are associated with the consumption of amino groups and the progress of the UTSA-MR [37]. New peaks were exhibited at 1323.892 cm^−1^, which indicated new substances were formed. These changes demonstrate that the conjugation occurred between CTS and JGI under the effect of UTSA-MR. As the proportion of CTS increases, the characteristic peaks of CTS-JGI are getting closer to CTS, which is caused by the masking of the characteristic peaks of JGI after the increase of CTS proportion.

#### 3.2.4 CD analysis

CD is a relatively simple and effective technique for studying the secondary structure of proteins [7]. The secondary structure distribution of CTS-JGI conjugates is predicated by CD and presented in Table S2. A significant decrease of α-helix and increase of β-sheet and random in the heated JGI and CTS-JG conjugates compared to the native JGI in the previous study with 54.11 % α-helix, 18.60 % β-sheet, and 27.29 % other structures [18]. These changes in the JGI secondary structures indicating a significant change in JGI spatial structure after heating and glycosylation. The reaction of the polysaccharide carbonyl group with the protein amino group is usually in the α-helix region and its surrounding structures. The decrease in the α-helix ratio shows that the helical region is the central region of the reaction. Besides, ultrasound can also influence the secondary structure ratios considering its effect on exposing the interior amino acids to the surface. The same result also appeared in Li’s study that α-helix of peanut protein decreased after conjugating with gum Arabic and dextran through ultrasonic treatment [22].

#### 3.2.5 Surface hydrophobicity (H_0_)

The surface hydrophobicity of proteins refers to folding proteins in aqueous media that tends to bury hydrophobic residues inside the molecule [21]. The balance of hydrophobic and hydrophilic interactions plays a vital role in protein structure and function. The H_0_ values of CTS-JGI conjugates are shown in Table S3. The H_0_ values of CTS-JGI-0.5, CTS-JGI-1, and CTS-JGI-2 were significantly (*p* < 0.05) higher than the heated JGI, which was mainly caused by the hydrophobic peptides’ exposure to the surface of JGI and the addition of polysaccharides tends to compete with ANS, thereby inhibiting the binding of ANS to protein groups. However, the lowest H_0_ appeared in CTS-JGI-4 due to the steric hindrance of CTS [54]. Meanwhile, as the proportion of CTS increases, the surface hydrophobicity tends to decrease in the four conjugates. This phenomenon is due to the binding of hydrophilic linear polysaccharides in CTS increasing the hydrophilicity of the conjugates molecular surface, thereby reducing the exposure of buried hydrophobic groups within the molecule. A study showed that the surface hydrophobicity of proteins was related to α-helix [44]. The link between α-helix and H_0_ is again demonstrated by the reduction of the helix structure and H_0_ value after the reaction in this experiment. This reduction is because glycosylation loosens the structure of JGI, allowing it to cross-link with CTS.

#### 3.2.6 SEM observation

The morphology of the JGI and CTS-JGI conjugates is shown in Fig. 3E-H. JGI exhibited a smoother and stiffer form. In contrast, the conjugates after the UTSA-MR showed a cross-networking structure. This result is ascribed to the changes in JGI structure after glycosylation with CTS. The Maillard reaction at high temperatures is beneficial to drawing polysaccharides and proteins, thereby improving the efficiency of the grafting reaction. This improvement of the reaction may be due to the high temperature that unfolds the protein structure and accelerates molecular movement, promoting the covalent interaction of the protein with the polysaccharides.

#### 3.2.7 Amino acid composition

An amino acid analyzer was used to analyze the amino acid composition of JGI to explore the changes in the amino acid composition of JGI during the reaction. The result is exhibited in Table 1. The total amount of amino acids in the conjugate is significantly reduced compared to native JGI, indicating that the reaction between CTS and JGI consumes the amino acids of JGI. Besides, the proportion of hydrophobic amino acids in the conjugate decreased while the proportion of hydrophilic amino acids increased. Pro, Ala, Val, Ile, Leu, Phe, Ser, Tyr, Lys, His, and Arg content significantly decreased, demonstrating that these amino acids are potential binding sites with CTS. A content increase of Met, Gly, and Cys appeared, which contributed to the unfolding of JGI structure, exposing these internal amino acids. Moreover, Thr, Asp, and Glu had no significant content change. One reason is that they are not involved in the reaction. Another deduction is that they are consumed in the same amount exposed from the internal structure during the reaction. Maillard reaction does not increase the total amount of essential amino acids. Only the content of Met significantly (*p* < 0.01) increased.

**Table 1:** Amino acid composition of JGI and CTS-JGI conjugate.

	Amino acids	Content (mg/g)	
		JGI	CTS-JGI
Hydrophile			
	Asp	17.72 ± 1.26	18.60 ± 1.44
	Ser	12.95 ± 1.18	10.12 ± 0.74*
	Glu	48.23 ± 1.99	43.24 ± 2.14
	Gly	11.38 ± 0.95	29.37 ± 1.98^**^
	Cys	0.97 ± 0.034	13.08 ± 0.78^**^
	Tyr	25.63 ± 1.41	13.78 ± 1.11^**^
	**Lys**	9.26 ± 0.69	4.61 ± 0.20^***^
	**His**	8.48 ± 0.79	4.61 ± 0.31^**^
	Arg	23.00 ± 1.57	8.07 ± 0.49^***^
	Total	157.62 ± 6.98	145.48 ± 3.30*

Hydrophobicity			
	**Thr**	10.79 ± 0.44	9.38 ± 0.58
	Pro	23.83 ± 1.72	17.93 ± 1.21*
	Ala	22.64 ± 1.56	16.47 ± 1.10*
	**Val**	17.92 ± 1.23	11.58 ± 0.21^***^
	**Met**	3.14 ± 0.15	19.75 ± 1.54^**^
	**Ile**	13.61 ± 0.81	9.67 ± 0.63^**^
	**Leu**	33.97 ± 2.15	26.04 ± 1.45*
	**Phe**	27.78 ± 1.52	15.01 ± 1.02^**^
	Total	153.38 ± 10.25	125.83 ± 7.74^***^

Values are means ± standard deviation; significant different between CTS-JGI and JGI was expressed as *, p < 0.05 and **, p < 0.01, and ***, p < 0.001. Bold amino acids are essential amino acids in human body.

### 3.3 CTS-JGI physicochemical properties after UTSA-MR

#### 3.3.1 Solubility analysis

The solubility of the conjugates is shown in Fig. 3I. Four conjugates have high solubility. This increase in solubility is consistent with the fact that the Maillard reaction can improve the solubility of substances. This result may ascribe to the isoelectric point of CTS as opposite to JGI. The covalent bonding of two substances changes the isoelectric point of cross-linker, thereby preventing the aggregation of CTS and JGI and improving the solubility. UTSA-MR leads to the unfolding of the protein, cleavage of peptide bonds, and exposure of hydrophilic amino acid residues at the internal sites of the protein, which also leads to an increase in the solubility of JGI [22]. However, when the proportion of CTS increased, the solubility of the complex decreased. This phenomenon is due to the CTS at a high proportion is not combined with JGI, and the isoelectric point of this part of the CTS does not change and cannot be dissolved.

#### 3.3.2 Foaming property and stability

The foaming property and stability are shown in Fig. 3J. The foaming property of CTS-JGI-1 and CTS-JGI-2 is the highest and significantly higher than native JG measured in our laboratory [18]. The foaming stability in the four conjugates was higher than native JG, in which CTS-JGI-2 exhibited the highest stability. This result indicates that the UTSA-MR can enhance the conjugate’s foaming property and stability under proper ratios between polysaccharides and proteins. This improvement in foamability and stability can be attributed to the increase in crosslinker solubility and random structure content [45].

#### 3.3.3 Emulsification activity index and emulsification stability index

The formation of protein-polysaccharide conjugates combines protein adsorption properties at the oil–water interface and the solvation properties of polysaccharides in aqueous media. Therefore, glycation could enhance the emulsifying property of the conjugates [49]. The EAI and ESI of CTS-JGI conjugates are shown in Table 2. As expected, the conjugates EAI and ESI were significantly (*p* < 0.05) increased compared with heated JGI. These results can explain that the UTSA-MR promote the stretching of protein molecules, enhance the attachment of more proteins to the polysaccharide and reduce the tension, thus improving the emulsion performance [45]. Therefore, CTS is an ideal polysaccharide to improve the emulsifying properties of JGI through protein–polysaccharide graft reactions. A study reports that the random structure in protein contributes to better emulsion properties [51]. This result is proved by the increased random ratio in CTS-JGI conjugates.

**Table 2:** EAI and ESI of heated JGI and CTS-JGI conjugates.

Conjugates	EAI	ESI
Heated JGI	37.96 ± 3.25^e^	225.80 ± 11.21^e^
CTS-JGI-0.5	197.00 ± 18.61^a^	658.62 ± 16.73^d^
CTS-JGI-1	179.98 ± 18.47^b^	1124.44 ± 28.00^b^
CTS-JGI-2	169.70 ± 4.44^c^	866.04 ± 24.46^c^
CTS-JGI-4	145.06 ± 4.67^d^	1268.04 ± 38.54^a^

Values are means ± standard deviation; values with different letters within a column differ significantly (p < 0.05).

#### 3.3.4 Viscosity

The viscosity of CTS-JGI conjugates is exhibited in Table S4. The viscosity of the CTS-JGI conjugates was increased significantly (*p* < 0.05) compared to native JGI in the previous study [18], which was ascribed to the combination with CTS and the increase of the conjugate solubility. Pirestani’s study also reported that the Maillard reaction could improve the viscosity of canola protein isolate with gum Arabic conjugate owing to the significant effect of gum Arabic addition and formation of a new macromolecule after covalent conjugation [28]. However, the viscosity of the four CTS-JGI conjugates did not show regularity, and there was no significant difference in viscosity.

#### 3.3.5 Thermal stability

It has been studied that the Maillard reaction can improve the thermal stability of proteins and their mixtures [26]. DSC can detect changes in the heat flow of the sample during temperature changes, which can provide beneficial information about the thermal stability of the sample. [54]. The thermal stability of JGI and CTS-JGI conjugates was presented in Fig. S1-S3. Compared with JGI, the fastest weight loss rate was at 280 ℃, significantly higher than JGI at 248 ℃. This result indicates that the UTSA-MR could enhance the thermal stability of CTS-JGI conjugates. Meanwhile, ΔH values in the conjugates significantly decreased compared to JGI. This result is consistent with a previous study that lower ΔH represents better thermal stability [54]. This stability is mainly attributed to the opening of the steric structure and increase repulsion forces between JGI molecules after the UTSA-MR and combining with CTS, resulting in a more stable secondary structure.

### 3.4 Stability of CTS-JGI oil-in-water nano-emulsions during storage

#### 3.4.1 Particle size, polydispersity index (PDI), and zeta-potential measurement

**Fig. 4:**
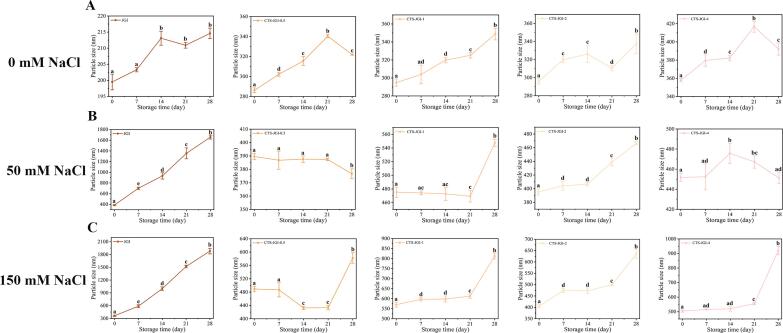
Particle size changes of the CTS-JGI conjugates during storage with (A) 0, (B) 50, and (C)150 mM NaCl solutions. Different letters showed a significant difference at *p* < 0.05.

**Fig. 5:**
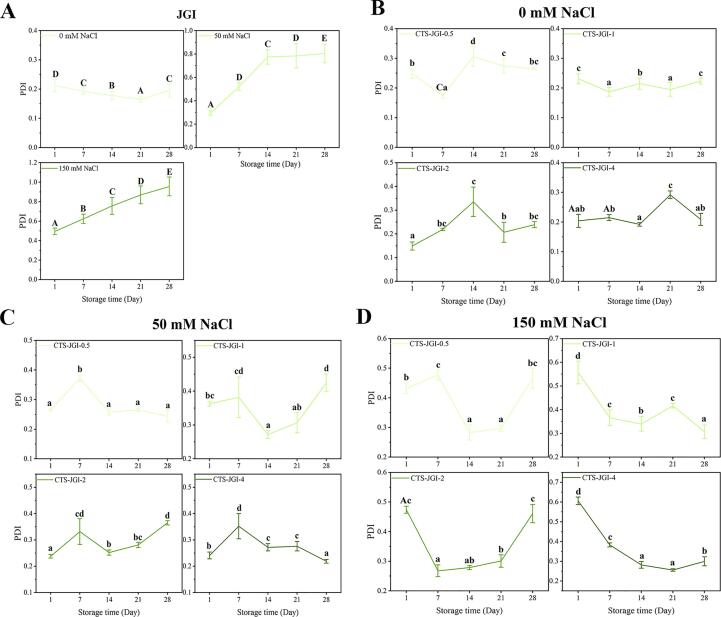
PDI changes of the (A) JGI and CTS-JGI conjugates during storage with (B) 0, (C) 50, and (D)150 mM NaCl solutions. Different letters showed a significant difference at *p* < 0.05.

**Fig. 6:**
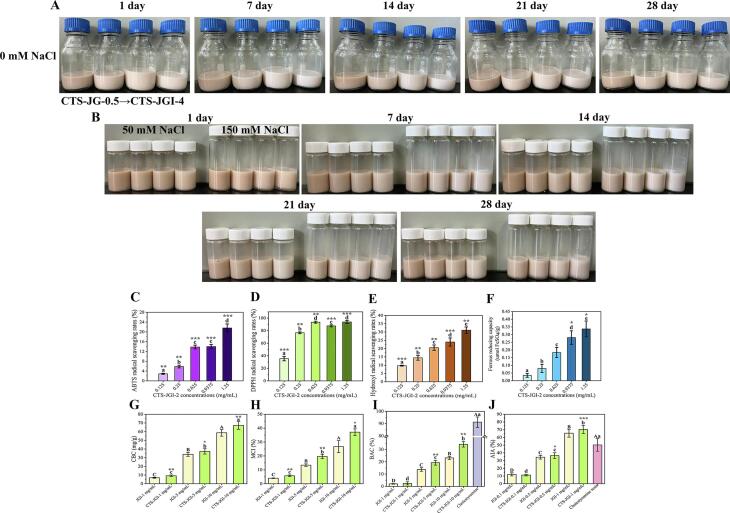
(A) Visual appearance of CTS-JGI conjugates stabilize oil-in-water nano-emulsions during storage. (B) Visual appearance of CTS-JGI conjugates stabilize oil-in-water emulsions during storage with 50 and 150 mM NaCl solutions. *In vitro* antioxidant abilities of CTS-JGI-2 conjugate. (C) ABTS, (D) DPPH, and (E) hydroxyl radical scavenging abilities. (F) ferrous reducing ability. *In vitro* cholesterol-lowering abilities. (G) CBC. (H) MCI. (I) BAC. (J) AIA. Different letters showed a significant difference at *p* < 0.05. *, *p* < 0.05, **, *p* < 0.01, and ***, *p* < 0.001 means significant difference between JGI in the previous study/in this study and CTS-JGI-2.

The particle size, PDI, zeta-potential, and graphs of the nano-emulsions were measured. As shown in Fig. 4A, 5A-B, Table S5, and Fig. 6A, the particle size remained at 286.2–357.9 nm when the emulsions were formed. The particle size increased with the ratios of CTS due to the viscosity of CTS, causing slight aggregation in the emulsions. Moreover, CTS-JGI-2 exhibited the lowest PDI, indicating stability in the emulsions. The same result existed that CTS-JGI-2 possessed the highest zeta-potential with a value of −28.05 mV. These results suggest that emulsifying ability of JGI can be positively affected by conjugating with CTS. Due to their improved solubility and viscosity, the CTS-JGI conjugates can be absorbed quickly on the oil-in-water interface.

To investigate the stability of CTS-JGI stabilized oil-in-water nano-emulsions. The particle sizes, PDI, and emulsion states are tracked continuously during 28 days of storage (Fig. 4A, 5B, and 6A). An increase in particle size can be observed during storage, possibly due to the coagulation of particles in the emulsion. The particle size in the four conjugate-formed emulsions increased by 34.3–53.8 nm during the storage period because of the aggregation, while the change in PDI was not noticeable.

#### 3.4.2 Backscattering intensity and Turbiscan scan index analysis

To further explore the stability of CTS-JGI oil-in-water nano-emulsions, BSI and TSI were measured to analyze the destabilization mechanisms. The x- and y-axes represent the height of the tube and the percentage change in BSI from the initial stage, respectively. The differently colored lines in the profile represent the change in BSI of the emulsion at different storage times. It is worth mentioning that from the BSI profile, four emulsions seem to produce stable BSI values, which is consistent with visual observations (Fig. S4A). The migration of oil droplets caused by the increase in buoyancy and the decrease in gravity may be the reason for the instability of the emulsion. This phenomenon may be caused by the fact that the oil droplets are not covered by protein with a prolonged storage time [33]. However, the BSI value does not appear obviously in the figure, indicating that the emulsion is relatively stable. Among the four emulsions, the CTS-JGI-0.5 and CTS-JGI-2 stabilized emulsions had the lowest BSI value under 40. Coagulation appeared at the bottom of the emulsion during 28-day storage. A study has shown that precipitation and flocculation are not significant factors affecting the physical properties of nano-emulsions, as they are prevalent in these emulsions [32]. This aggregation phenomenon may be caused by the increase in particle diameter and decrease in particle concentration due to coalescence or Ostwald ripening [31].

TSI is another crucial indicator reflecting the stability of the emulsion. In general, a lower TSI value means better stability of the emulsion. As shown in Fig. S4B, the TSI values of the emulsions’ global, bottom, middle, and top parts were measured. TSI values in different parts of the emulsions remained at low values, indicating the stability of the emulsions. However, the top part exhibited a high TSI value. This phenomenon is caused by the flocculation and aggregation of the low-density oil phase in the upper layer of the emulsions. Overall, CTS-JGI-2 had the lowest TSI at different positions of the emulsions.

#### 3.4.3 Stability of emulsions against NaCl

The presence of electrolytes in the emulsion makes the system unstable. To determine the ability of the CTS-JGI conjugates to resist ions in the stabilized emulsions, NaCl was added to the emulsion at concentrations of 50 mM and 150 mM. The particle size, PDI changes, and emulsion graphs are shown in Fig. 4B-C, 5C-D, and 6B. The particle size and PDI increased when NaCl was added to the emulsions. This phenomenon is because the addition of ions reduces the electrostatic repulsion between particles, and the system becomes unstable so that particles aggregate to form stable large particles [25]. The emulsion particle size in the 50 mM NaCl groups increased less in the following measurements. The emulsion particle size even became smaller in CTS-JGI-1 and CTS-JGI-2. In the 150 mM groups, the initial particle size of the emulsions increased significantly (*p* < 0.05), but the change was not apparent in the following 21 days. On the 28th day, both groups significantly (*p* < 0.05) increased the particle size and PDI, but PDI remained within a stable range during storage. The PDI decreased in the CTS-JGI stabilized oil-in-water emulsions, indicating that CTS-JGI could resist NaCl-induced agglomeration.

The above emulsion stabilization experiments conclude that CTS-JGI-2 possesses the best stabilization effect on the emulsion. Therefore, it is selected for the following functional experiments.

### 3.5 Functional properties of CTS-JGI

#### 3.5.1 *In vitro* antioxidant activities

The hydroxyl radical is a highly reactive oxygen species. The hydroxyl radical scavenging rate is an important index to evaluate the free radical scavenging ability [34]. Therefore, the scavenging activity against hydroxyl radicals is one of the most effective protections against diseases caused by free radical-induced oxidative stress. ABTS and DPPH free radical scavenging abilities are also critical indicators to measure the antioxidant capacity of functional components. The *in vitro* antioxidant abilities of CTS-JGI-2 are shown in Fig. 6C-F. The same concentrations of JGI in the previous study were used to evaluate the four assays [18]. CTS-JGI-2 exhibited ABTS, DPPH, and hydroxyl scavenging abilities, and the abilities increased with the concentrations. The DPPH ability of CTS-JGI-2 was the highest among the three radicals. This increase in DPPH ability is due to the Maillard reaction producing new peptides in the low molecular weight region with enhanced DPPH radical scavenging ability. These peptides are hydrophobic and can combine with DPPH radicals, thus showing a stronger scavenging power. In addition, the improvement of the free radical scavenging activity of CTS-JGI-2 is related to the chromogenic groups, deoxyfructose, pyrrolidones, and reducing ketones generated during the reaction [47]. The hydrogen atoms of these antioxidant molecules are combined with the free radicals of the DPPH molecules and stabilize the molecule without chain reactions. This high DPPH radical scavenging ability is precisely consistent with the result of the increased H_0_. However, the ferrous reducing ability of CTS-JGI-2 did not significantly increase in the concentrations of 0.125–0.625 mg/mL compared to JGI. The ferrous reducing power increased only at the two high concentrations. Usually, free hydroxyl groups play an essential role in the ferrous force, which can provide hydrogen atoms that destroy the free radical chain and increase the reducing power activity [38]. From this, it can be speculated that CTS-JGI-2′s radical scavenging ability is stronger than ferrous reducing power.

#### 3.5.2 Cholesterol-lowering activities

Many mechanisms explain the cholesterol-lowering functional effect of related components. One of the most accepted explanations is that the ingestion of these components disturbs the enterohepatic circulation, thereby causing the excretion of diseased cholesterol and bile acids. The cholesterol-lowering effect of protein-based components is related to their insolubility. Indigestible proteins or polypeptides rich in hydrophobic amino groups (which can combine with bile acids) are post-digested or fecal. The cholesterol-lowering abilities of JGI and CTS-JGI-2 were firstly determined. As shown in Fig. 6G-J, three concentrations of JGI and CTS-JGI-2 exhibited cholesterol-binding capacity (7.46 ± 0.54–62.58 ± 3.88 mg/g and 10.12 ± 0.96–71.52 ± 4.41 mg/g). The cholesterol-binding capacity of CTS-JGI-2 is significantly higher than JGI. This observation confirms that Maillard reaction conjugates have a comparable potential to bind to cholesterol directly. The inhibiting cholesterol micellar solubility is critical in reducing intestinal absorption of dietary cholesterol. JGI showed MCI values of 4.21 ± 0.21–31.03 ± 4.29 %, while higher MCI values appeared in CTS-JGI-2 (6.32 ± 0.54–39.48 ± 2.38 %).

Bile acids are biosynthesized from endogenous cholesterol metabolism. If bile acids in the intestinal lumen bind to sequestering agents, such as cholestyramine, enterohepatic circulation will be disrupted, and bile acid reabsorption and endogenous cholesterol levels are reduced [14]. Similar to the above index results, it can be observed that the UTSA-MR remarkably (*p* < 0.01) increased the bile acid-binding ability in the 5 and 10 mg/mL of CTS-JGI-2 compared to native JGI (increased by 5.55 % and 11.04 %, respectively). However, the bile acid-binding potential of CTS-JGI-2 is remarkably lower than cholestyramine (the positive control in this study).

α-amylase converts carbohydrates of food into sucrose, which can be consumed by the body as energy or stored as lipid [42]. α-amylase inhibitory activity plays an essential role in cholesterol-lowering, and inhibition of α-amylase activity can hinder the digestion and absorption of starch and other carbohydrates in food [35]. It can be seen from the result that both JGI and CTS-JGI-2 exhibited α-amylase inhibitory activity. The 0.5 and 1 mg/mL of CTS-JGI-2 showed a higher α-amylase inhibitory activity than native JGI (increased by 2.14 % and 4.82 %, respectively). Although the inhibition rate was higher than that of cholestyramine resin, the concentration of CTS-JGI-2 was much higher. Thus, the α-amylase inhibitory activity of CTS-JGI-2 does not reach the inhibitory ability of the standard drug.

### 3.6 Encapsulation efficiency and loading capacity

**Fig. 7:**
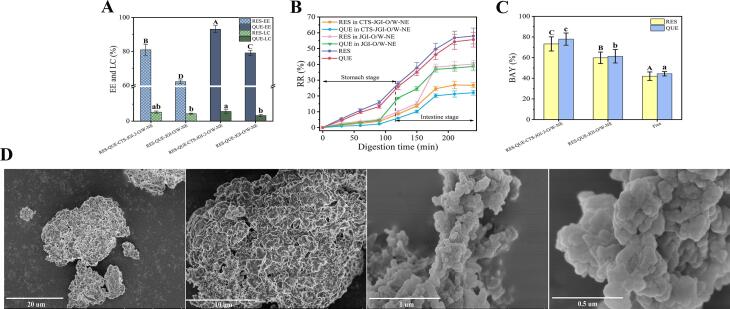
(A) EE and LC of RES and QUE in CTS-JGI-2-O/W-PE and JGI- O/W-PE. (B) RR of RES and QUE in CTS-JGI-2-O/W-PE, JGI- O/W-PE, and free RES and QUE during *in vitro* digestion. (C) BAY of RES and QUE in CTS-JGI-2-O/W-PE, JGI- O/W-PE, and free RES and QUE. Different letters showed a significant difference at *p* < 0.05. (D) Microstructure of RES/QUE-CTS-JGI-2 particles at different resolutions.

To estimate the protective effect of CTS-JGI-2 on active substances, RES and QUE were loaded in the CTS-JGI-2-O/W-NE. As illustrated in Fig. 7A, the EE of RES and QUE loaded in CTS-JGI-2-O/W-NE increased significantly (*p* < 0.05) than JGI, which was ascribed to the enhancement of loading ability after the UTSA-MR. However, the LC showed no significant different between the two emulsions, which was caused by the high LC leading to lower EE at overload [53]. The EE and LC of QUE were significantly (*p* < 0.05) higher than RES, proving a better loading ability of QUE in CTS-JGI-2 and JGI. This better loading ability is contributed to the stable binding effect between QUE with CTS-JGI-2 and JGI. The same result was also obtained in Zhang’s study that the Maillard reaction product of whey protein and flaxseed gum also increased the EE and maintained the LC of astaxanthin [53].

### 3.7 *In vitro* digestion of emulsions

The release rates of RES and QUE in the emulsions are exhibited in Fig. 7B. It could be seen that the CTS-JGI-2-O/W-NE was resistant to pepsin hydrolysis due to the low release rate compared to JGI-O/W-NE (0–120 min). However, the release rates of RES and QUE remained under 10 %. This resistance to pepsin hydrolysis may be due to the formation of a stable bond between CTS-JGI-2 and JGI with RES and QUE so that a large steric hindrance appears between pepsin with RES and QUE. This phenomenon results in a slower release of RES and QUE because pepsin needs to hydrolyze the outer CTS-JGI-2 or JGI before releasing RES and QUE. The digestion process of pepsin is primarily limited by the surface area and erosion rate of the CTS-JGI-2 particles in the emulsion. RES and QUE in CTS-JGI-2-O/W-NE have a slower digestion rate than JGI-O/W-NE, and it is speculated that the emulsion covalently linked to CTS through the UTSA-MR can shield the cleavage site of pepsin by JGI [53], [12].

When the emulsions were transferred to the intestinal condition, the RR increased significantly in the first 1 h. The increase of RR is contributed to the better solubility of JGI in alkaline environment provided by intestinal fluid, enabling the release of RES and QUE. The RR remained stable at 180–240 min. After the digestion, the total RRs were 57.96 % of RES, 55.69 % of QUE, 40.2 % of RES-JGI-O/W-NE, 38.69 % of QUE-JGI-O/W-NE, 26.77 % of RES-CTS-JGI-2-O/W-NE, and 22.06 % of QUE-CTS-JGI-2-O/W-NE, respectively. As shown in Fig. 7C, the BAY of RES and QUE in JGI-O/W-NE (59.8 % for RES and 61.31 % for QUE) and CTS-JGI-2-O/W-NE (73.23 % for RES and 77.94 % for QUE) were significantly (*p* < 0.05) higher than RES (42.04 %) and QUE (44.34 %) alone. Worthy to note that the BAY of QUE in this study was significantly higher than rice bran protein-based nano-emulsion in Chen’s study [7]. The above results suggest that CTS-JGI-2 improves the bioavailability of RES and QUE.

Furthermore, to preliminarily determine the structural changes of CTS-JGI-2 after encapsulation of RES and QUE. The microstructure of RES and QUE-loaded CTS-JGI-2 structures is shown in Fig. 7D. The particles have a dense networked structure. As the resolution increases, the particle path becomes elliptical. This shape is not significantly different from that of the CTS-JGI alone. A study [3] has shown that temperature during freeze-drying affects the shape of the particles, while the rapid sublimation of water frozen during freeze-drying from the matrix can lead to the formation of cavities where ice crystals previously existed, often resulting in structures with rough and shrunken surfaces. However, the surface of CTS-JGI obtained in this experiment formed a dense network of cross-links, indicating that its structural stability was not affected by freeze-drying.

**Fig. 8:**
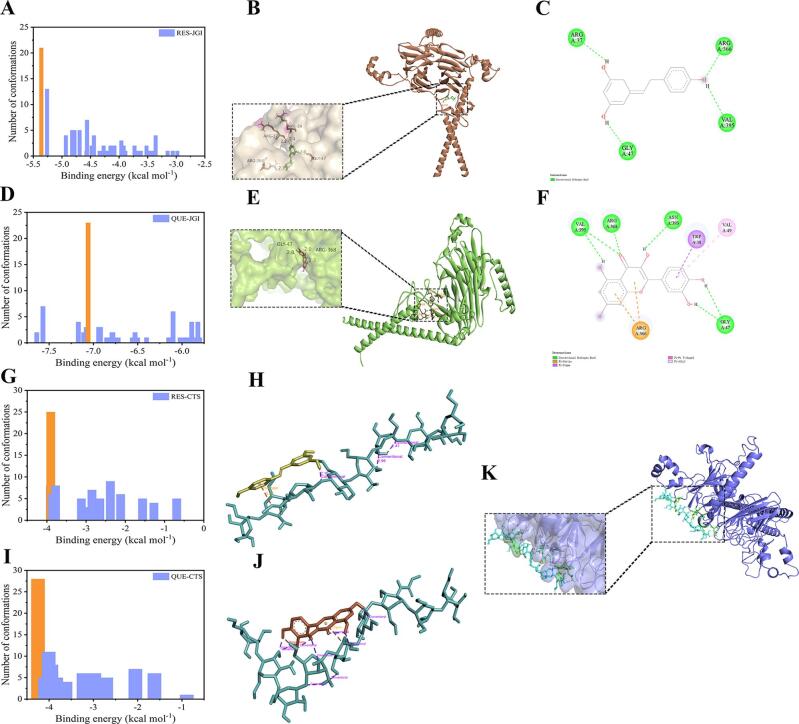
(A, D, G, and I) Cluster analysis of docking runs. (B and E) Three-dimensional potential binding sites between RES and QUE with JGI. (C and F) Two-dimensional potential binding sites between RES and QUE with JGI. (H and J) Three-dimensional potential binding sites between RES and QUE with CTSU. (K) Three-dimensional potential binding sites between CTSU with JGI.

In addition, molecular docking is used to deeply explore the binding mechanism between RES and QUE with CTS-JGI-2. After molecular docking simulation, the conformation clusters with the highest number of times and lowest binding energy were regarded as the possible binding region (Fig. 8A and D). Fig. 8B, C, E, and F reveals the potential binding sites between RES and QUE with JGI in the CTS-JGI-2. RES could bind with JGI through hydrogen bonds at Arg-37, Gly-47, Arg-366, and Val-395. QUE could combine with JGI through hydrogen bonds at Gly-47, Arg-368, Asn-393, and Val-395; pi-cation at Arg-366; pi-sigma at Trp-38; and pi-alkyl at Val-49, respectively. The binding energy of QUE with JGI (-7.06 kcal mol^−1^) is lower than that of RES with JGI (-5.36 kcal mol^−1^), and binding sites of QUE with JGI exceed RES with JGI, which indicates that the binding of QUE with JGI is more stable than RES. The possible bindings between RES and QUE with CTS were also investigated. Considering the complexity of the CTS structure, the CTSU was selected for the docking simulation. The binding between RES (-3.90 kcal mol^−1^) and QUE (-4.25 kcal mol^−1^) with CTSU required higher energy than the bindings with JGI (Fig. 8G and I). The binding sites are shown in Fig. 8H and J. Hydrogen bond and pi-anion appeared in the binding between RES with CTSU. More hydrogen bonds, pi-sigma, and donors contributed to the binding between QUE with CTSU compared to RES. More stable combinations between QUE with JGI and CTSU correspond to the higher EE, LC, RR, and BAY of QUE than RES. However, RES and QUE have the same docking site as JGI (Gly-47). Considering that the ratio of RES and QUE is much lower than JGI, there is no competitive docking relationship between the two substances.

Moreover, the potential binding between CTSU and JGI was also predicted. The potential binding sites are shown in Fig. 8K. The binding energy is positive (data not shown), indicating the combination needs energy from the outside and cannot spontaneously combine. UTSA-MR provides the energy for the conjugation between JGI and CTS, so they can finally combine.

## 4 Conclusion

In conclusion, JGI was combined with CTS through UTSA-MR to obtain the conjugates with better solubility, thermal stability, emulsifying stability, antioxidant ability, and cholesterol-lowering activity. This improvement might attribute to the JGI structural modification by the reaction with CTS. Furthermore, the O/W-NE stabilized by CTS-JGI-2 was preeminent for entrapment and site-specific release of RES and QUE. The above results highlight that the covalent linkage of JGI and CTS through UTSA-MR can be an ideal method to improve its bioactive properties and expand its utilization in the food nano-emulsion stabilization and functional substances delivery system. More investigation on the *in vivo* protection of RES- and QUE-loaded nano-emulsion stabilized by CTS-JGI-2 needs to be carried out in further study.

## CRediT authorship contribution statement

**Yunsong Jiang:** Conceptualization, Methodology, Software, Formal analysis, Data curation, Writing – original draft, Investigation, Validation, Writing – review & editing. **Kai Zang:** Data analysis. **Jinyuan Sun:** Funding acquisition, Supervision, Writing – review & editing. **Xin-an Zeng:** Writing – review & editing. **Hehe Li:** Resources. **Charles Brennan:** Writing – review & editing. **Mingquan Huang:** Writing – review & editing. **Ling Xu:** Resources.

## Declaration of Competing Interest

The authors declare that they have no known competing financial interests or personal relationships that could have appeared to influence the work reported in this paper.

## References

[1] Abd El-Salam M.H., El-Shibiny S., Salem A. (2009). Factors Affecting the Functional Properties of Whey Protein Products: A Review. Food Rev. Int..

[2] Akande K.E., Doma U.D., Agu H.O., Adamu H.M. (2010). Major Antinutrients Found in Plant Protein Sources: Their Effect on Nutrition. Pak. J. Nutr..

[3] Al-Maqtari Q.A., Mohammed J.K., Mahdi A.A., Al-Ansi W., Zhang M.i., Al-Adeeb A., Wei M., Phyo H.M., Yao W. (2021). Physicochemical properties, microstructure, and storage stability of Pulicaria jaubertii extract microencapsulated with different protein biopolymers and gum arabic as wall materials. Int. J. Biol. Macromol..

[4] Bai L., Huan S., Rojas O.J., McClements D.J. (2021). Recent Innovations in Emulsion Science and Technology for Food Applications. J. Agric. Food. Chem..

[5] Caddeo C., Pons R., Carbone C., Fernàndez-Busquets X., Cardia M.C., Maccioni A.M., Fadda A.M., Manconi M. (2017). Physico-chemical characterization of succinyl chitosan-stabilized liposomes for the oral co-delivery of quercetin and resveratrol. Carbohydr. Polym..

[6] Chen P., Shen Z., Ming L., Li Y., Dan W., Lou G., Peng B.o., Wu B., Li Y., Zhao D.a., Gao G., Zhang Q., Xiao J., Li X., Wang G., He Y. (2018). Genetic Basis of Variation in Rice Seed Storage Protein (Albumin, Globulin, Prolamin, and Glutelin) Content Revealed by Genome-Wide Association Analysis. Front. Plant Sci..

[7] Chen W., Ju X., Aluko R.E., Zou Y., Wang Z., Liu M., He R. (2020). Rice bran protein-based nanoemulsion carrier for improving stability and bioavailability of quercetin. Food Hydrocolloids.

[8] Chen X., Fang F., Wang S. (2020). Physicochemical properties and hepatoprotective effects of glycated Snapper fish scale peptides conjugated with xylose via maillard reaction. Food Chem. Toxicol..

[9] Du Y.-L., Huang G.-Q., Wang H.-O., Xiao J.-X. (2018). Effect of high coacervation temperature on the physicochemical properties of resultant microcapsules through induction of Maillard reaction between soybean protein isolate and chitosan. J. Food Eng..

[10] Fatease A.A., Shah V., Nguyen D.X., Cote B., LeBlanc N., Rao D.A., Alani A.W.G. (2019). Chemosensitization and mitigation of Adriamycin-induced cardiotoxicity using combinational polymeric micelles for co-delivery of quercetin/resveratrol and resveratrol/curcumin in ovarian cancer. *Nanomedicine: Nanotechnology*. Biol. Med..

[11] Han S.-W., Chee K.-M., Cho S.-J. (2015). Nutritional quality of rice bran protein in comparison to animal and vegetable protein. Food Chem..

[12] Huo J., Wu Z., Sun W., Wang Z., Wu J., Huang M., Wang B., Sun B. (2022). Protective Effects of Natural Polysaccharides on Intestinal Barrier Injury: A Review. J. Agric. Food Chem..

[13] Jaisamut P., Limsuwan S., Chusri S., Wiwattanapatapee R., Wiwattanawongsa K. (2020). Enhanced Oral Bioavailability and Improved Biological Activities of a Quercetin/Resveratrol Combination Using a Liquid Self-Microemulsifying Drug Delivery System. Planta Med..

[14] Jia W., Xie G., Jia W. (2018). Bile acid–microbiota crosstalk in gastrointestinal inflammation and carcinogenesis. Nat. Rev. Gastroenterol. Hepatol..

[15] Jiang W., Zhang Y., Julian McClements D., Liu F., Liu X. (2022). Impact of pea protein-inulin conjugates prepared via the Maillard reaction using a combination of ultrasound and pH-shift treatments on physical and oxidative stability of algae oil emulsions. Food Res. Int..

[16] Jiang Y., Sun J., Yin Z., Li H., Sun X., Zheng F. (2020). Evaluation of antioxidant peptides generated from Jiuzao (residue after Baijiu distillation) protein hydrolysates and their effect of enhancing healthy value of Chinese Baijiu. J. Sci. Food Agric..

[17] Jiang Y., Wang R., Yin Z., Sun J., Wang B., Zhao D., Zeng X.-a., Li H., Huang M., Sun B. (2021). Optimization of Jiuzao protein hydrolysis conditions and antioxidant activity in vivo of Jiuzao tetrapeptide Asp-Arg-Glu-Leu by elevating the Nrf2/Keap1-p38/PI3K-MafK signaling pathway. Food Funct..

[18] Jiang Y., Xing M., Kang Q., Sun J., Zeng X.A., Gao W., Li A. (2022). Pulse electric field assisted process for extraction of Jiuzao glutelin extract and its physicochemical properties and biological activities investigation. Food Chem..

[19] Karbasi M., Sánchez-Ferrer A., Adamcik J., Askari G., Madadlou A., Mezzenga R. (2021). Covalent β-lactoglobulin-maltodextrin amyloid fibril conjugate prepared by the Maillard reaction. Food Chem..

[20] Klinchongkon K., Khuwijitjaru P., Adachi S., Bindereif B., Karbstein H.P., van der Schaaf U.S. (2019). Emulsifying properties of conjugates formed between whey protein isolate and subcritical-water hydrolyzed pectin. Food Hydrocolloids.

[21] Kumar A., Venkatesu P. (2012). Overview of the Stability of α-Chymotrypsin in Different Solvent Media. Chem. Rev..

[22] Li C., Xue H., Chen Z., Ding Q., Wang X. (2014). Comparative studies on the physicochemical properties of peanut protein isolate–polysaccharide conjugates prepared by ultrasonic treatment or classical heating. Food Res. Int..

[23] Li W., Wang C., Yuan G., Pan Y., Chen H. (2018). Physicochemical characterisation and α-amylase inhibitory activity of tea polysaccharides under simulated salivary, gastric and intestinal conditions. Int. J. Food Sci. Technol..

[24] Li Z., Zheng Y., Sun Q., Wang J., Zheng B., Guo Z. (2021). Structural characteristics and emulsifying properties of myofibrillar protein-dextran conjugates induced by ultrasound Maillard reaction. Ultrason. Sonochem..

[25] McClements D.J., Gumus C.E. (2016). Natural emulsifiers — Biosurfactants, phospholipids, biopolymers, and colloidal particles: Molecular and physicochemical basis of functional performance. Adv. Colloid Interface Sci..

[26] Nasrollahzadeh F., Varidi M., Koocheki A., Hadizadeh F. (2017). Effect of microwave and conventional heating on structural, functional and antioxidant properties of bovine serum albumin-maltodextrin conjugates through Maillard reaction. Food Res. Int..

[27] Pacheco-Quito E.-M., Ruiz-Caro R., Veiga M.-D. (2020). Carrageenan: Drug Delivery Systems and Other Biomedical Applications. Mar. Drugs.

[28] Pirestani S., Nasirpour A., Keramat J., Desobry S., Jasniewski J. (2017). Effect of glycosylation with gum Arabic by Maillard reaction in a liquid system on the emulsifying properties of canola protein isolate. Carbohydr. Polym..

[29] Raghavendra N., Hublikar L.V., Chitnis R.S., A Joseph R., Sheelimath S.D., Pattan S.P. (2020). Areca catechu seed extract as improvised acid-base indicator in titrimetric Analysis: An environmental benign approach. J. Water Environ. Nanotechnol..

[30] Rodríguez-Vázquez M., Vega-Ruiz B., Ramos-Zúñiga R., Saldaña-Koppel D.A., Quiñones-Olvera L.F. (2015). Chitosan and Its Potential Use as a Scaffold for Tissue Engineering in Regenerative Medicine. BioMed Res. Int..

[31] Saberi A.H., Fang Y., McClements D.J. (2013). Effect of glycerol on formation, stability, and properties of vitamin-E enriched nanoemulsions produced using spontaneous emulsification. J. Colloid Interface Sci..

[32] Shamekhi M.A., Rabiee A., Mirzadeh H., Mahdavi H., Mohebbi-Kalhori D., Baghaban Eslaminejad M. (2017). Fabrication and characterization of hydrothermal cross-linked chitosan porous scaffolds for cartilage tissue engineering applications. Mater. Sci. Eng.: C.

[33] Shi J., Xiao J., Liu L., Dong X. (2021). Ultrasonic assisted oil-in-water emulsions stabilized by flaxseed protein isolate: influence of different oils. J. Dispersion Sci. Technol..

[34] Singh H.P., Kaur S., Mittal S., Batish D.R., Kohli R.K. (2010). In vitro screening of essential oil from young and mature leaves of Artemisia scoparia compared to its major constituents for free radical scavenging activity. Food Chem. Toxicol..

[35] Sun L., Wang Y., Miao M. (2020). Inhibition of α-amylase by polyphenolic compounds: Substrate digestion, binding interactions and nutritional intervention. Trends Food Sci. Technol..

[36] Tan J.E., Liu T., Yao Y., Wu N., Du H., Xu M., Tu Y. (2021). Changes in physicochemical and antioxidant properties of egg white during the Maillard reaction induced by alkali. LWT.

[37] Umemura K., Kawai S. (2007). Modification of chitosan by the Maillard reaction using cellulose model compounds. Carbohydr. Polym..

[38] Valko M., Jomova K., Rhodes C.J., Kuča K., Musílek K. (2016). Redox- and non-redox-metal-induced formation of free radicals and their role in human disease. Arch. Toxicol..

[39] Vapor A., Mendonça A., Tomaz C.T. (2022). Processes for reducing egg allergenicity: Advances and different approaches. Food Chem..

[40] Wan Z.-L., Guo J., Yang X.-Q. (2015). Plant protein-based delivery systems for bioactive ingredients in foods. Food Funct..

[41] Wang C., Wang M., Zhang M.-P. (2021). Ethyl carbamate in Chinese liquor (Baijiu): presence, analysis, formation, and control. Appl. Microbiol. Biotechnol..

[42] Wang S., Chen L., Yang H., Gu J., Wang J., Ren F. (2020). Regular intake of white kidney beans extract (Phaseolus vulgaris L.) induces weight loss compared to placebo in obese human subjects. Food Sci. Nutr..

[43] Wang W., Meng Q., Li Q., Liu J., Zhou M., Jin Z., Zhao K. (2020). Chitosan Derivatives and Their Application in Biomedicine. Int. J. Mol. Sci..

[44] Wang Z., Li Y., Jiang L., Qi B., Zhou L. (2014). Relationship between Secondary Structure and Surface Hydrophobicity of Soybean Protein Isolate Subjected to Heat Treatment. J. Chem..

[45] Wen C., Zhang J., Qin W., Gu J., Zhang H., Duan Y., Ma H. (2020). Structure and functional properties of soy protein isolate-lentinan conjugates obtained in Maillard reaction by slit divergent ultrasonic assisted wet heating and the stability of oil-in-water emulsions. Food Chem..

[46] Widyarani, Bowden N.A., Kolfschoten R.C., Sanders J.P.M., Bruins M.E. (2016). Fractional Precipitation of Amino Acids from Agro-industrial Residues Using Ethanol. Ind. Eng. Chem. Res..

[47] Xiong G.Y., Chen X., Zhang X.X., Miao Y., Zou Y., Wang D.Y., Xu W.M. (2020). Process optimization and the relationship between the reaction degree and the antioxidant activity of Maillard reaction products of chicken liver protein hydrolysates. Poult. Sci..

[48] Xu L., Geelen D. (2018). Developing Biostimulants From Agro-Food and Industrial By-Products.

[49] Xu Y., Dong M., Tang C., Han M., Xu X., Zhou G. (2020). Glycation-induced structural modification of myofibrillar protein and its relation to emulsifying properties. LWT.

[50] Xu Z.-Z., Huang G.-Q., Xu T.-C., Liu L.-N., Xiao J.-X. (2019). Comparative study on the Maillard reaction of chitosan oligosaccharide and glucose with soybean protein isolate. Int. J. Biol. Macromol..

[51] Yang F., Liu X., Ren X., Huang Y., Huang C., Zhang K. (2018). Swirling cavitation improves the emulsifying properties of commercial soy protein isolate. Ultrason. Sonochem..

[52] Yang X., Li Y., Li S., Ren X., Olayemi Oladejo A., Lu F., Ma H. (2020). Effects and mechanism of ultrasound pretreatment of protein on the Maillard reaction of protein-hydrolysate from grass carp (Ctenopharyngodon idella). Ultrason. Sonochem..

[53] Zhang Z., Chen W., Zhou X., Deng Q., Dong X., Yang C., Huang F. (2021). Astaxanthin-loaded emulsion gels stabilized by Maillard reaction products of whey protein and flaxseed gum: Physicochemical characterization and in vitro digestibility. Food Res. Int..

[54] Zhong L., Ma N., Wu Y., Zhao L., Ma G., Pei F., Hu Q. (2019). Characterization and functional evaluation of oat protein isolate-Pleurotus ostreatus β-glucan conjugates formed via Maillard reaction. Food Hydrocolloids.

[55] Zhu L., Song X., Pan F., Tuersuntuoheti T., Zheng F., Li Q., Sun B. (2021). Interaction mechanism of kafirin with ferulic acid and tetramethyl pyrazine: Multiple spectroscopic and molecular modeling studies. Food Chem..

[56] Zhu W.-W., Tang C.-H. (2022). Mild preheating improves cholesterol-lowering benefits of soy protein via enhancing hydrophobicity of its gastrointestinal digests: An in vitro study. Food Hydrocolloids.

