# Supplementary material for: Preparation of modified Jiuzao glutelin isolate with carboxymethyl chitosan by ultrasound-stirring assisted Maillard reaction and its protective effect of loading resveratrol/quercetin in nano-emulsion
Source: Ultrason Sonochem. 2022 Jul 12;88:106094. doi: 10.1016/j.ultsonch.2022.106094 (PMC9305625; doi:10.1016/j.ultsonch.2022.106094)
Supplement: Supplementary data 6 [file mmc6.docx]

**Preparation of modified Jiuzao glutelin isolate with carboxymethyl chitosan by ultrasound-stirring assisted Maillard reaction and its protective effect of loading resveratrol/quercetin in nano-emulsion**

Yunsong Jiang^a,b^, Kai Zang^a^, Jinyuan Sun^a,*^, Xin-an Zeng^b^, Hehe Li^a^, Charles Brennan^c^, Mingquan Huang^a^, Ling Xu^d^, Baoguo Sun^a^

^a^Key Laboratory of Brewing Molecular Engineering of China Light Industry, Beijing Technology and Business University, Beijing 100048, People’s Republic of China

^b^School of Food Science and Engineering, South China University of Technology, Guangzhou, People’s Republic of China

^c^School of Science, RMIT, Melbourne, VIC 3000, Australia

^d^Technology Center of Bandaojing Co. Ltd., Zibo, Shandong, 256300, People’s Republic of China

- 1. **Materials and methods**
  2. Materials and regents

BCA protein assay kit, o-phthalaldehyde (OPA), BCA assay kit, DNS assay kit, and SDS were obtained from Beijing Solarbio Science & Technology Co., Ltd. (Beijing, China). Furfural (standard for GC, 99.5%) was purchased from Aladdin Biochemical Technolgoy Co., Ltd (Shanghai, China). ABTS kit was bought from Nanjing Jiancheng Institute of Biotechnology (Nanjing, China). DPPH, ferrous reducing, and hydroxyl assay kits were purchased from Shanghai HuicH Biotech Co., Ltd (Shanghai, China). Carboxymethyl chitosan (CTS, water solubility), α-amylase, 5, 5’-dithiiobis-(2-nitrobenzoic acid) (DTNB), 8-Anilino-1-napthalene sulfonic acid (ANS), bile acid sodium, sodium doiocholate, anhydrous sodium triphosphate, oleic acid, starch, pepsin, trypsin and other analytical grade chemicals and solvents were bought from Macklin Biochemical Co., Ltd (Shanghai, China).

2.3 Determination of the pH changes, browning index (BI), A_294_, grafting degree (GD), and surface disulfide bond content

The pH changes of the CTS-JGI system during the Maillard reaction were measured by a pH meter SevenExcellence S400 (METTLER TOLEOO, Zurich, Switzerland).

For BI measurement, the absorbance of the CTS-JGI samples obtained after different reaction times was measured using an automated microplate reader (Molecule Device, Silicon Valley, CA, USA) at 420 nm.

For intermediate product measurement, the CTS-JGI samples obtained after different reaction times were diluted in ultrapure water to a concentration of 1 mg/mL. The absorbance was measured at 294 nm using an automated microplate reader.

For surface disulfide bond content measurement, a DTNB reaction solution was prepared according to a previous study (Jiang et al., 2022) by mixing 10 mM DTNB, 86 mM Tris-HCl, 90 mM glycine, and 4 mM EDTA Na_2._ 2.5 mg/mL of CTS-JGI solutions were prepared using pH 7 PBS. 20 μL of DTNB solution was added to 200 μL of CTS-JGI solutions and kept for 15 min at 25 ℃ without light. JGI was used as the blank. The absorbance was measured at 412 nm. The surface sulfhydryl content change percent was calculated according to the following formula:

$\text{Sulfhydryl content change (\%)=(}\frac{\text{S}\text{t}\text{-S}\text{0}}{\text{S}\text{0}}\text{)×100\%}$ (1)

where S_t_ is the absorbance of the CTS-JGI after reactions and S_0_ is the absorbance of heat-treated JGI.

2.4.5 Determination of surface hydrophobicity (H_0_)

20 μL of the 8-anilino-1-naphthalenesulfonic acid (ANS) solution (8 mM, pH 7) was added to the CTS-JGI solutions. After mixing, the fluorescence intensity of the samples was recorded by a microplate reader at an excitation wavelength of 390 nm and an emission wavelength of 470 nm. H_0_ was calculated by the initial slope of the fluorescence intensity and JGI concentrations curve.

- 1. Physicochemical properties
     1. Solubility

After mixing and centrifugation, the protein concentration of the supernatant was measured by using BCA method according to the instruction of the assay kit. The solubility of the conjugates was calculated by the following formula:

$\text{Solubility (\%)=(}\frac{\text{Protein concentration in supernatant}}{\text{Total protein concentration in the sample}}\text{)×100\%}$ (2)

- - 1. Foaming property and stability

The foaming properties of CTS-JGI conjugates were measured according to the method of (Nasrollahzadeh, Varidi, Koocheki, & Hadizadeh, 2017) with slight modification. 5 mg/mL of sample solution was placed in a messcylinder. The volume of the sample was recorded as A_1_. The sample solution was homogenized by using a homogenous machine (IKA T25 digital, Baden-Wuerttemberg. German) at a speed of 15,000 rpm for 1 min. The volume was recorded as A_2_. After 15 min, the sample volume was recorded again as A_3_. The foaming ability and stability were calculated by the following equations:

$\text{Foaming ability (\%)=(}\frac{\text{A}\text{2}\text{-A}\text{1}}{\text{A}\text{1}}\text{)×100\%}$ (3)

$\text{Foaming stability (\%)=(}\frac{\text{A}\text{3}\text{-}\text{A}\text{1}}{\text{A}\text{1}}\text{)×100\%}$ (4)

- - 1. Emulsification activity index (EAI) and emulsification stability index (ESI)

EAI and ESI of CTS-JGI conjugates were investigated according to the method of (Chen et al., 2019) with slight modification. 14 mL of the sample solutions (5 mg/mL) were mixed with 6 mL of soybean oil. The solution was mixed using a homogenous machine (IKA T25 digital, Baden-Wuerttemberg. German) at 16,000 rpm for 1 min. After the emulsion was formed, the EAI and ESI were calculated according to Chen’s (Chen et al., 2019) method. Briefly, 40 μL of the emulsions were mixed with 4 mL of 0.1% SDS solution and vortexed for 10 s (A_0_). The emulsions after 10 min (t) of silence were mixed with 0.1% SDS (A_10_). The absorbance was read at 500 nm. 0.1% SDS solution was used as the blank. The EAI and ESI were determined using the following equations, respectively:

$\text{EAI (}\frac{\text{m}\text{2}}{\text{g}}\text{)=(}\frac{\text{2}\text{×2.303×}\text{A}\text{0}}{\text{5}\text{×0.3×100}}\text{)}$ (5)

$\text{ESI (}\text{min}\text{)=(}\frac{\text{A}\text{0}}{\text{A}\text{0}\text{-A}\text{10}}\text{)×10}$ (6)

- - 1. Viscosity

The viscosity of the samples (2.67 mg/mL) was determined by using a BROOKFIELD VISCOMETER Z2210-0389 (MA, USA) at 25 ℃. The measurement conditions were listed in Table S3.

- - 1. Thermal stability

Samples (3 mg) were placed in an aluminum pan. After sealing, the temperature was raised from 25 ℃ to 600 ℃ at a heating rate of 10 ℃/min. The thermal stability of the conjugates was measured by using thermogravimetry (TG)-different scanning calorimetry (DSC) (TA Q200 and TA DSC25, New Castle, USA).

- - 1. Cholesterol-lowering activities

For cholesterol-binding capacity (CBC) measurement, 3 mL of the sample (10 mg/mL) was mixed with 7.5 mL of the yolk dispersion and 4.5 mL of ultrapure water. After the reaction, 40 μL of the concentrate solution was mixed with 360 μL acetic acid, 1.5 mL of o-phthalaldehyde (OPA) reagent, and 1 mL of sulfuric acid. After 20 min reaction, the absorbance was read at 550 nm. The cholesterol concentration was calculated by the standard curve prepared by the cholesterol solution. CBC was calculated as follows:

$\text{CBC (mg/g)=(}\frac{\text{(C}\text{2}\text{-C}\text{3}\text{)-(C}\text{1-}\text{C}\text{2}\text{)}}{\text{M}}\text{)×10}$ (7)

where C_1_, C_2_, and C_3_ are the concentrations of cholesterol in the yolk, the yolk without samples, and the yolk mixed with samples (mg/mL), respectively; 10 is the adsorption volume (mL), and M is the dry weight of the sample (g).

For micellar cholesterol inhibition (MCI) measurement, 20 μL of conjugate (10 mg/mL) was added to 180 μL of the emulsion. After vortexing, the mixture was incubated for 1 h at 37 ℃. The supernatant was obtained by centrifugation at 8000 *g* for 15 min. The content of cholesterol was determined by the OPA method. MCI was calculated according to the following formula:

$\text{MCI (\%)=(}\frac{\text{C}\text{0}\text{-C}\text{s}}{\text{C}\text{0}}\text{)×100\%}$ (8)

where C_0_ is the original micelle cholesterol concentration and C_s_ is the sample micelle cholesterol concentration.

For bile acid-binding capacity (BAC) assays, 0.1 mL of the sample solution (10 mg/mL in pH 7.4 PBS) was mixed with 0.9 mL of bile acid sodium (2 mM in pH 7.4 PBS). After incubation for 2 h at 37 ℃. The resultant mixture was centrifuged at 12,000 *g* for 12 min to obtain the supernatant. Then, 0.4 mL of the concentrate solution was mixed with 2.4 mL of 45% sulfuric acid and 0.4 mL of 0.1% furfural. The absorbance was read at 620 nm. Cholestyramine was used as the positive control. Bile acids without samples were used as blank control. The BAC of JGI and CTS-JGI was calculated using the following equation:

$\text{BAC (\%)=(}\frac{\text{A}\text{sample}\text{-A}\text{blank}}{\text{A}\text{blank}}\text{)×100\%}$ (9)

For α-amylase inhibitory activity (AIA) determination, 100 μL of sample solution (1 mg/mL) was mixed with 200 μL α-amylase and reacted for 5 min at 37 ℃. Afterward, 500 μL of 1 wt% starch solution was added and reacted for 5 min at 37 ℃. Subsequently, 500 μL of DNS solution was added to the mixture and reacted for 5 at 100 ℃. Finally, 40 μL of the reaction mixture was diluted by 160 μL 0.1 M PBS (pH 6.8). Cholestyramine resin was used as the positive control and PBS (pH 6.8) was regarded as the blank control. The absorbance was measured at 540 nm. The inhibitory rate was measured by the equation as follows:

$\text{AIA}\text{ (\%)=(}\frac{\text{1-A}\text{s}}{\text{A}\text{C}}\text{)×100\%}$ (10)

where A_S_ is the absorption of the samples and A_C_ is the absorption of the blank control.

**Reference**

Chen, W., Ma, X., Wang, W., Lv, R., Guo, M., Ding, T., . . . Liu, D. (2019). Preparation of modified whey protein isolate with gum acacia by ultrasound maillard reaction. *Food Hydrocolloids, 95*, 298-307. doi:<https://doi.org/10.1016/j.foodhyd.2018.10.030>

Jiang, Y., Xing, M., Kang, Q., Sun, J., Zeng, X. A., Gao, W., . . . Li, A. (2022). Pulse electric field assisted process for extraction of Jiuzao glutelin extract and its physicochemical properties and biological activities investigation. *Food Chem, 383*, 132304. doi:10.1016/j.foodchem.2022.132304

Nasrollahzadeh, F., Varidi, M., Koocheki, A., & Hadizadeh, F. (2017). Effect of microwave and conventional heating on structural, functional and antioxidant properties of bovine serum albumin-maltodextrin conjugates through Maillard reaction. *Food Research International, 100*, 289-297. https://doi.org/10.1016/j.foodres.2017.08.030


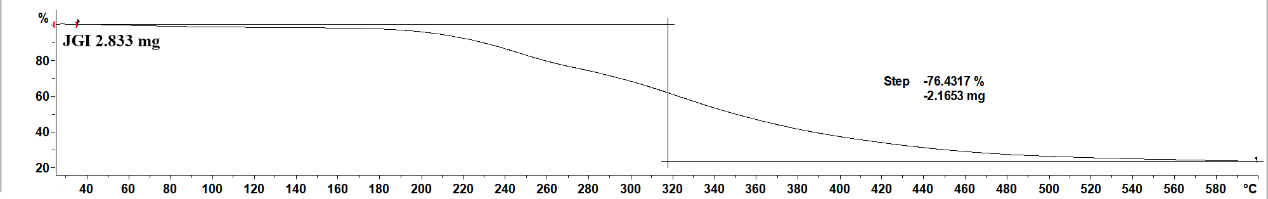


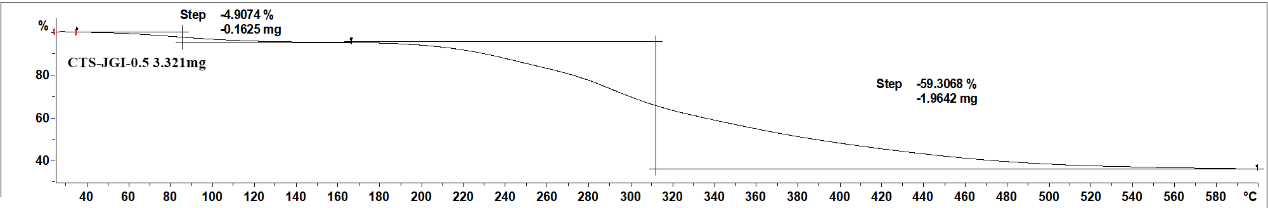


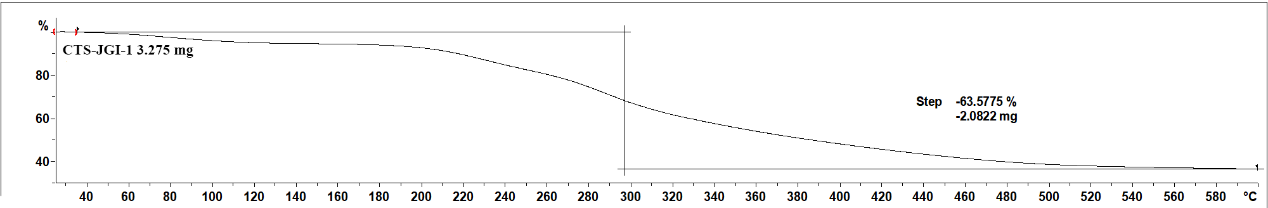


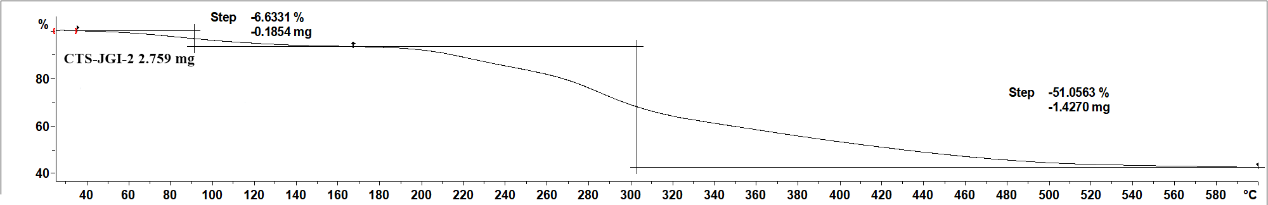


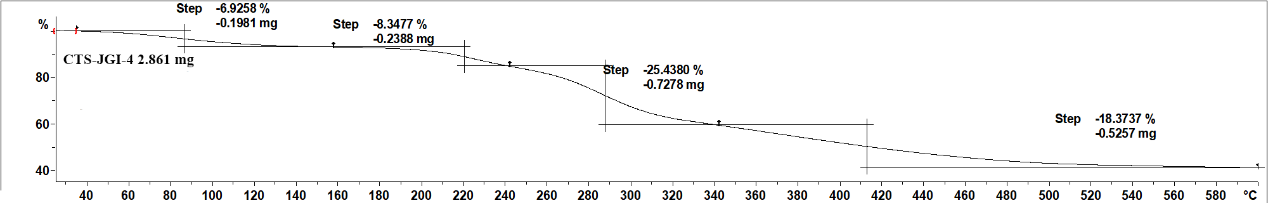


**Fig. S1** Weight changes of JGI and CTS-JGI conjugates of thermal treatment


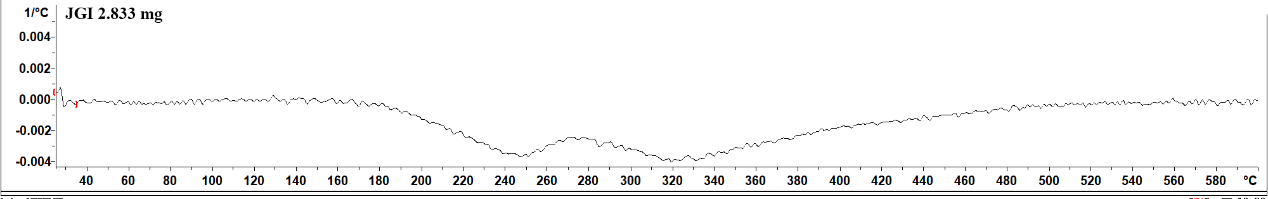


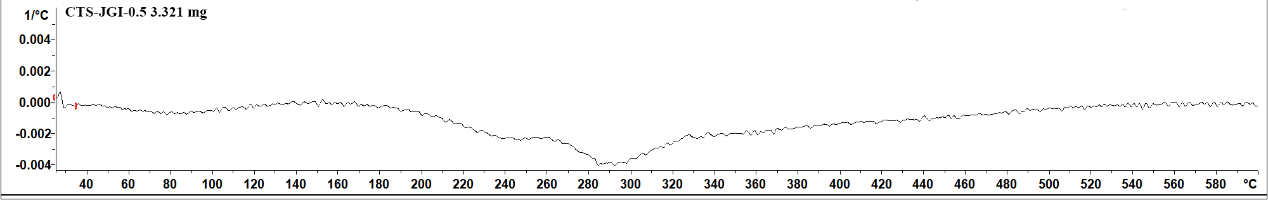


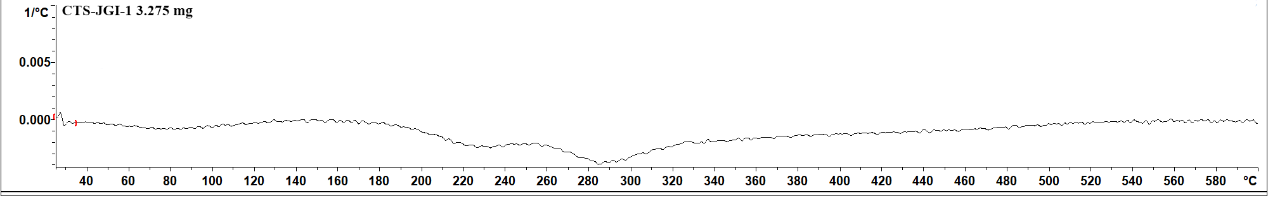


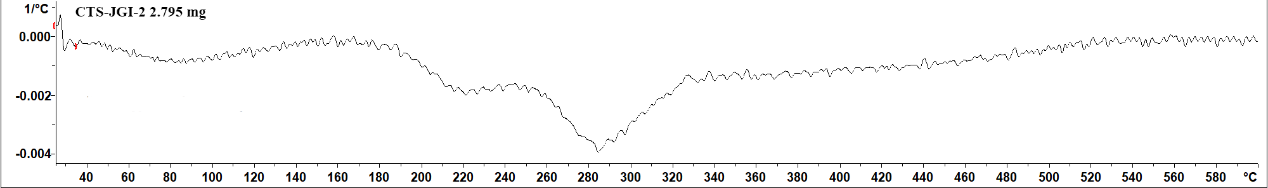


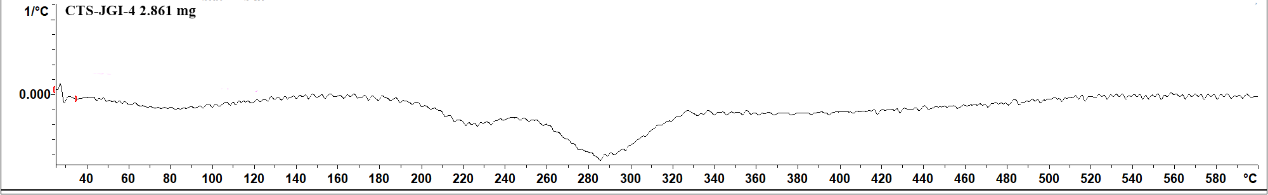


**Fig. S2** DTG of JGI and CTS-JGI conjugates of thermal treatment


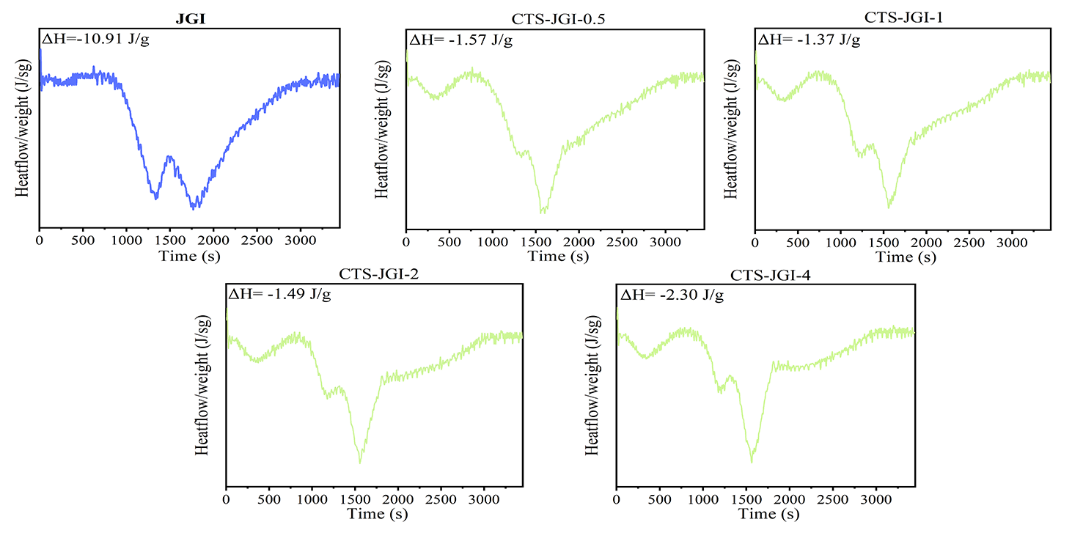


**Fig. S3** ΔH of JGI and CTS-JGI conjugates of thermal treatment.


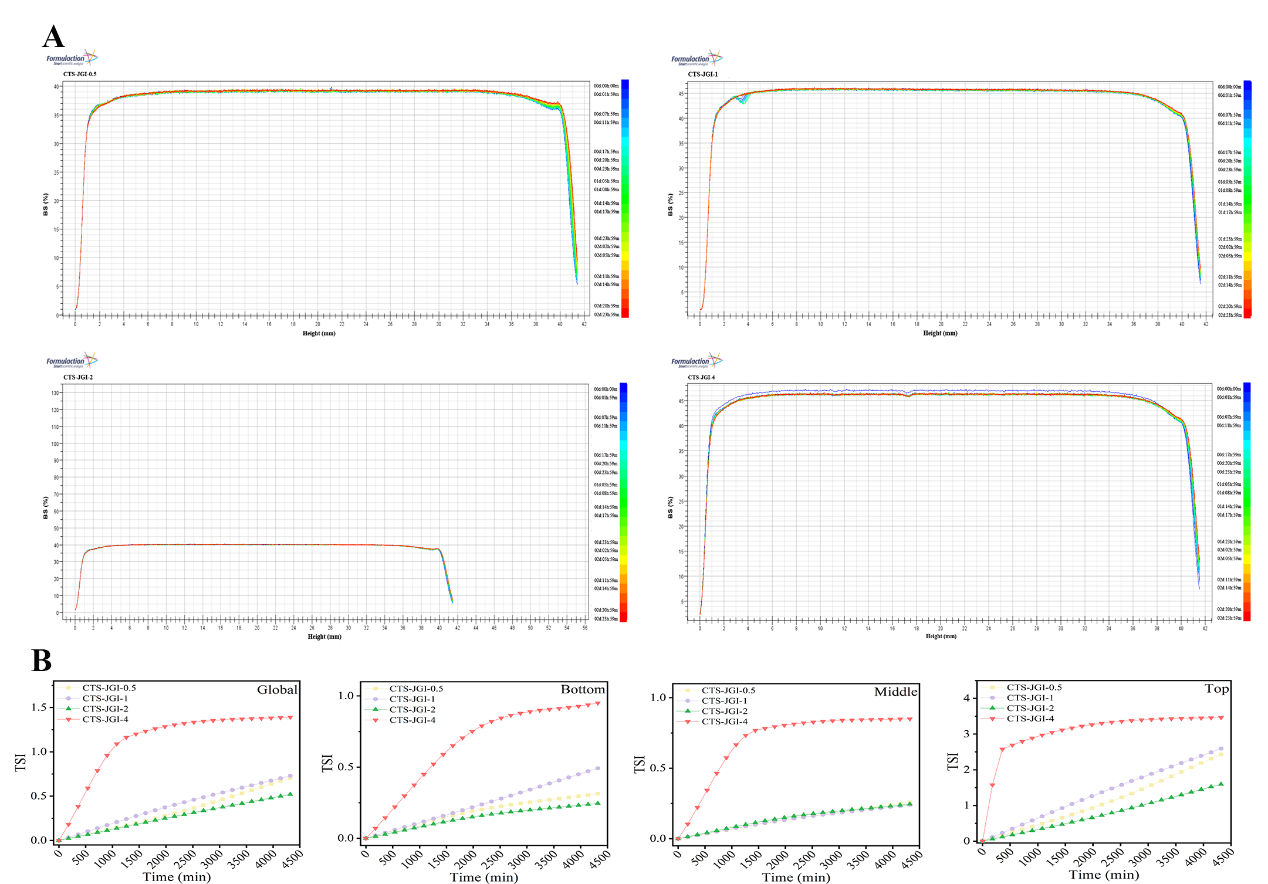


**Fig. S4** BSI and TSI of CTS-JGI conjugates. (A) BSI. (B) TSI.

**Table S1** JGI source and structure.

| Protein IDs | Predicted structure | Protein Match | Mw (kDa) |
| --- | --- | --- | --- |
| C5WUN6 | 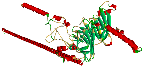 | Sorghum bicolor uncharacterized protein | 74.13 |
| A0A1D6L7G4; A0A1D6L7F8 | 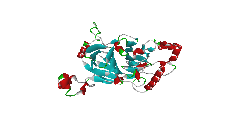 | Maize globulin | 47.37 |
| P0CZ08; P0CZ07 | 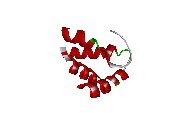 | Wheat avenin-like a3 | 19.33 |
| I6QQ39 | 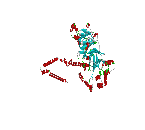 | Wheat Globulin | 66.32 |
| T1T4Y4; A1YQG3 | 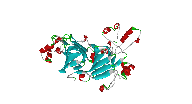 | Oyrsi Glutelin | 51.58 |

**Table S2** Secondary structure composition of CTS-JGI conjugates measured by CD.

| Conjugates | Alpha helix (%) | Beta sheet (%) | Random (%) |
| --- | --- | --- | --- |
| Heated JGI | 3 | 51 | 46 |
| CTS-JGI-0.5 | 4 | 48 | 48 |
| CTS-JGI-1 | 4.33 | 47.67 | 48 |
| CTS-JGI-2 | 4.67 | 47.33 | 47.67 |
| CTS-JGI-4 | 5 | 47 | 48 |

**Table S3** H_0_ of JGI and CTS-JGIs.

|  | Reaction conditions | H_0_ |
| --- | --- | --- |
| Heated JGI | pH 7 for 180 min | 348.56^a^ |
| CTS-JGI-0.5 | pH 7 for 90 min | 788.813^e^ |
| CTS-JGI-1 | pH 7 for 180 min | 609.66^d^ |
| CTS-JGI-2 | pH 7 for 180 min | 396.431^c^ |
| CTS-JGI-4 | pH 7 for 180 min | 160.897^b^ |

Different letters showed a significant difference at *p* < 0.05.

**Table S4** Viscosity of CTS-JGI conjugates.

|  | Viscosity | Torque (%) | Rotation (rpm) | Shear force | Shear rate (1/s) |
| --- | --- | --- | --- | --- | --- |
| Water | 1.81^a^ | 50.6 | 168 | 3.71 | 205.5 |
| CTS-JGI-0.5 | 2.03^c^ | 50.0 | 148 | 3.67 | 181 |
| CTS-JGI-1 | 1.95^b^ | 50.6 | 156 | 3.71 | 190.8 |
| CTS-JGI-2 | 1.98^b^ | 51.4 | 156 | 3.77 | 190.8 |
| CTS-JGI-4 | 1.99^bc^ | 50.5 | 152 | 3.71 | 185.9 |

Different letters showed a significant difference at *p* < 0.05.

**Table S5** Zeta-potential of JGI and CTS-JGI conjugates.

| Conjugates | Zeta-potential (mV) |
| --- | --- |
| Heated JGI | -5.46±0.480^d^ |
| CTS-JGI-0.5 | -16.86±0.388^b^ |
| CTS-JGI-1 | -11.82±0.091^c^ |
| CTS-JGI-2 | -28.05±0.425^a^ |
| CTS-JGI-4 | -11.47±0.072^c^ |

Different letters showed a significant difference at *p* < 0.05.
